# The Role of the Nuclear Factor κB Pathway in the Cellular Response to Low and High Linear Energy Transfer Radiation

**DOI:** 10.3390/ijms19082220

**Published:** 2018-07-30

**Authors:** Christine E. Hellweg, Luis F. Spitta, Kristina Koch, Arif A. Chishti, Bernd Henschenmacher, Sebastian Diegeler, Bikash Konda, Sebastian Feles, Claudia Schmitz, Thomas Berger, Christa Baumstark-Khan

**Affiliations:** 1 German Aerospace Centre (DLR), Institute of Aerospace Medicine, Radiation Biology, Linder Höhe, D-51147 Köln, Germany; luis.spitta@dlr.de (L.F.S.); tkoch@mediomix.de (K.K.); arif.chishti@kibge.edu.pk (A.A.C.); bernd.henschenmacher@dlr.de (B.H.); sebastian.diegeler@dlr.de (S.D.); bikash.konda@dlr.de (B.K.); sebastian.feles@dlr.de (S.F.); claudia.schmitz@dlr.de (C.S.); thomas.berger@dlr.de (T.B.); christa.baumstark-khan@dlr.de (C.B.-K.); 2 The Karachi Institute of Biotechnology and Genetic Engineering, University of Karachi, Karachi 75270, Sindh, Pakistan

**Keywords:** nuclear factor κB, RelA, linear energy transfer, heavy ion, space mission, cytokines, chemokines, cellular radiation response

## Abstract

Astronauts are exposed to considerable doses of space radiation during long-term space missions. As complete shielding of the highly energetic particles is impracticable, the cellular response to space-relevant radiation qualities has to be understood in order to develop countermeasures and to reduce radiation risk uncertainties. The transcription factor Nuclear Factor κB (NF-κB) plays a fundamental role in the immune response and in the pathogenesis of many diseases. We have previously shown that heavy ions with a linear energy transfer (LET) of 100–300 keV/µm have a nine times higher potential to activate NF-κB compared to low-LET X-rays. Here, chemical inhibitor studies using human embryonic kidney cells (HEK) showed that the DNA damage sensor Ataxia telangiectasia mutated (ATM) and the proteasome were essential for NF-κB activation in response to X-rays and heavy ions. NF-κB’s role in cellular radiation response was determined by stable knock-down of the NF-κB subunit RelA. Transfection of a RelA short-hairpin RNA plasmid resulted in higher sensitivity towards X-rays, but not towards heavy ions. Reverse Transcriptase real-time quantitative PCR (RT-qPCR) showed that after exposure to X-rays and heavy ions, NF-κB predominantly upregulates genes involved in intercellular communication processes. This process is strictly NF-κB dependent as the response is completely absent in RelA knock-down cells. NF-κB’s role in the cellular radiation response depends on the radiation quality.

## 1. Introduction

Exposure to galactic cosmic radiation which consists of protons, α-particles and heavier nuclei is a major risk factor for long-term human space missions. On the International Space Station (ISS), the effective dose rate quantified by human phantom experiments amounted to 550–570 µSv/d inside the station and 690–720 µSv/d during extravehicular activities [1,2]. During a mission to Mars, astronauts will accumulate considerable doses of galactic cosmic radiation of about 1 Sv [3,4]. Due to mass limitations for spacecraft and production of secondary particles in shielding material, the radiation exposure cannot simply be avoided by radiation shielding of the spacecraft [5]. The potential space radiation risks are cancer and non-cancer effects such as cataract formation [6,7] and degenerative effects in the central nervous and cardiovascular system [8,9]. Furthermore, acute effects may arise after exposure to a solar particle event during a situation of insufficient shielding [10,11].

Although heavy nuclei only make up about 2% of the fluence in space, they will account for much of the biological consequences due to their higher linear energy transfer (LET) [12]. Traversal of heavy nuclei through cellular structures produces ionization tracks along their path, resulting in the formation of complex DNA damage [13] which elicits the DNA damage response (DDR) leading to several possible outcomes, such as cell cycle arrest, allocating additional time for repair of the damaged DNA, cellular senescence or different types of cell death, if the damage is too severe and cannot be repaired [14] (reviewed in Hellweg et al., IJPT, in press). The transcription factor Nuclear Factor κB (NF-κB) is involved e.g., in regulation of proliferation, immune system development and performance, inflammation and apoptosis [15]. The activation NF-κB by ionizing radiation [16] was discovered early after its first description [17]. Exposure to accelerated heavy ions (95 MeV/nucleon-MeV/n-Ar, LET 272 keV/µm) resulted in much stronger activation of NF-κB in human cells than X-ray exposure [18]. The NF-κB activation by energetic carbon ions (35 and 75 MeV/n, LET 73 and 33 keV/µm, respectively) was comparable to the effect induced by X-rays [19], suggesting a narrow peak in the LET dependence of NF-κB activation. This was confirmed by studies with a large set of heavy ions, covering a LET range of ~30–10,000 keV/µm, which revealed a maximal NF-κB activation by heavy ions in a LET range of ~91–272 keV/µm [20]. This LET range is highly relevant for space radiation exposure of astronauts [21]. Furthermore, during the last fifteen years, in addition to the canonical and alternative pathways of NF-κB activation, a direct pathway for its activation by genotoxic stress was discovered. This sub-pathway involves a nuclear to cytoplasmic shuttle that transports the information from radiation-induced DNA double strand breaks in the cell nucleus to the cytoplasm where NF-κB is sequestered in its inactive state [22,23]. The released NF-κB translocates to the nucleus and binds to κB DNA motifs (NF-κB response elements, NRE) initiating gene transcription. NRE have been identified in the promoter or enhancer regions of a number of growth factors, antiapoptotic molecules, cytokines and adhesion molecules involved in fibrosis and inflammation [24,25]. Our recent work showed that the expression level of several NF-κB-dependent cyto- and chemokines after heavy ion exposure follows a similar LET dependence as NF-κB activation itself [26].

As NF-κB is involved in regulation of apoptosis and immune and inflammatory responses, its activation may influence the outcome of a heavy ion exposed cell (cellular survival/death, cell cycle arrest, DNA repair) and therefore might be relevant for late effects which might occur after prolonged low-dose exposure during an interplanetary mission and might be potential pharmacological target for mitigation of the radiation response. The aim of this work was to analyze the role of the NF-κB pathway in the cellular response to exposure to space-relevant high-LET radiation.

In order to enable monitoring of radiation-induced NF-κB activation, a previously developed reporter cell line, HEK-pNF-κB-d2EGFP/Neo L2 [27], was used. This cell line harbors a plasmid which reports NF-κB-dependent transcriptional activation in response to various stimuli simply by expressing the destabilized variant of enhanced green fluorescent protein (d2EGFP) [28] when activated NF-κB binds to its synthetic promoter which contains four NRE tandem copies. By measuring the yields of green fluorescence of the reporter protein it is possible to quantify NF-κB-dependent transcriptional activation in response to stimuli.

To assess the role of NF-κB in the cellular response to heavy ion exposure, besides the use of chemical inhibitors, the expression of the NF-κB subunit p65/RelA was downregulated by stable transfection of the reporter cell line with a RelA short-hairpin RNA (shRNA RelA). Growth, cell cycle progression, survival and gene expression after ionizing radiation exposure (low- and high-LET) were compared for cells with normal and reduced RelA expression. Due to limited beam time availability, heavy ion experiments were restricted to high-dose rate exposures.

## 2. Results

The role of the NF-κB pathway in the cellular response to low and high-LET radiation was studied in human embryonic kidney cells.

### 2.1. Kinetics of NF-κB Activation

**Figure 1:**
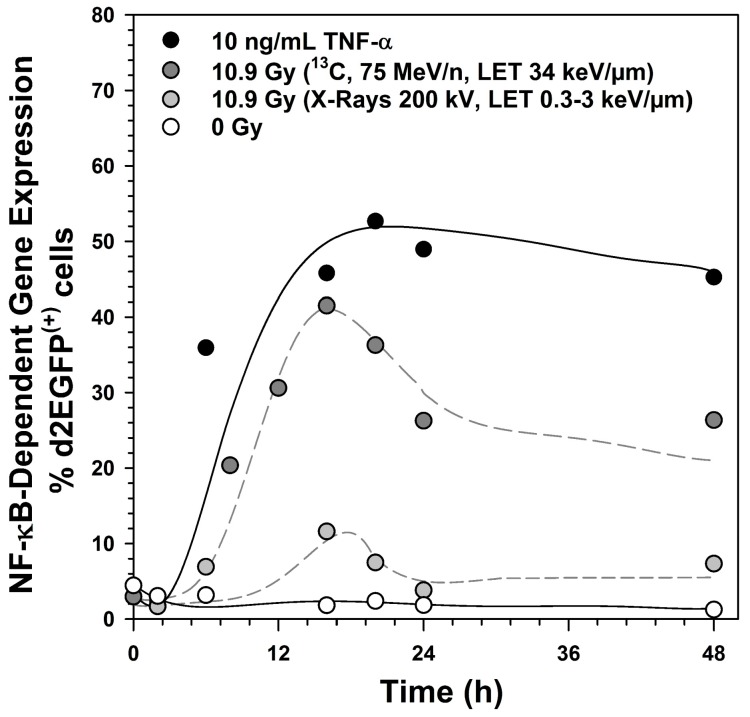
Kinetics of Nuclear Factor κB (NF-κB)-dependent destabilized enhanced green fluorescent protein (d2EGFP) expression after treatment with tumor necrosis factor α (TNF-α), X-rays and carbon ions, as detected in stably transfected human embryonic kidney cells (HEK) cells from d2EGFP reporter gene expression controlled by a promoter containing four NF-κB response elements (NRE) and the minimal promoter of thymidine kinase (TK) (HEK-pNF-κB-d2EGFP/Neo clone L2). HEK-pNF-κB-d2EGFP/Neo L2 cells were irradiated with X-rays (200 kV, linear energy transfer (LET) ~0.3–3 keV/µm) or ^13^C-ions (75 MeV/n, LET 34 keV/µm) or incubated with 10 ng/mL TNF-α as a positive control. At different time points after induction, cells were trypsinated and fixed with 3.7% formaldehyde. d2EGFP expression was analyzed by flow cytometry.

The kinetics of NF-κB-dependent expression of the reporter gene d2EGFP was determined after exposure to 10.9 Gy X-rays, 10.9 Gy carbon ions or 10 ng/mL tumor necrosis factor α (TNF-α) (Figure 1). The high dose was chosen as X-rays are a weak activator of NF-κB. The activation was strongest in response to TNF-α and reached a maximum after 20 h. After irradiation, the maximum was reached already after 16 h. Two days after carbon ion exposure and after addition of TNF-α, NF-κB dependent d2EGFP expression was still detectable in ~30% and ~50% of the cells, respectively. Activation by X-rays was transient and reached normal levels within one day.

### 2.2. Ataxia Telangiectasia Mutated (ATM) and the Proteasome Are Required for NF-κB Activation by X-rays and Heavy Ion Exposure

**Figure 2:**
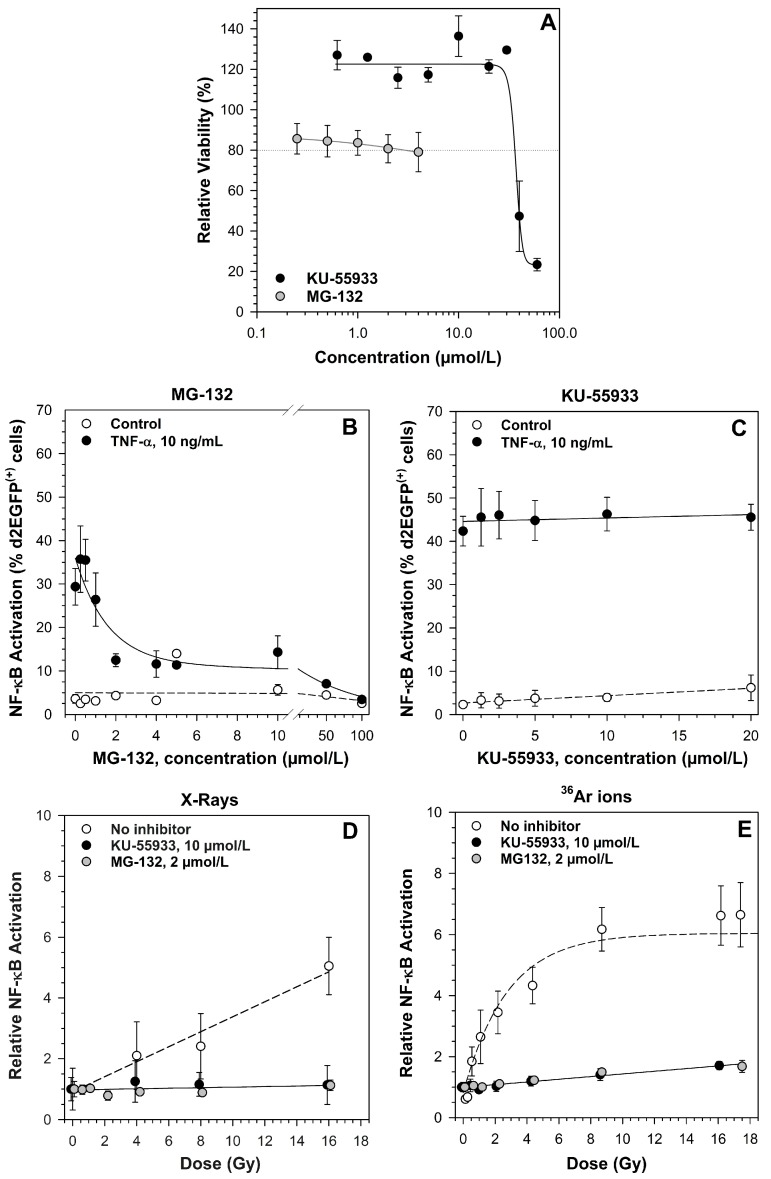
Ataxia telangiectasia mutated (ATM) and the proteasome are required for NF-κB activation by low and high LET radiation. Means and standard error (SE) of two to three independent experiments with three replicates each are shown. The viability of HEK-pNF-κB-d2EGFP/Neo L2 cells after treatment with increasing concentrations of ATM inhibitor KU-55933 and the proteasome inhibitor MG-132 was determined by means of the 3-(4,5-dimethylthiazole-2-yl-2,5-diphenyltetra- zolium bromide (MTT) test 100 h after adding the inhibitor. The dotted line indicates 80% relative viability (**A**); the effect of MG-132 (**B**) and KU-55933 (**C**) on TNF-α-induced activation of the NF-κB pathway was determined in HEK-pNF-κB-d2EGFP/Neo L2 cells preincubated with the inhibitor for 1 h before addition of 10 ng/mL TNF-α. Cells were detached and fixed with 3.7% formaldehyde after 18 h. NF-κB-dependent d2EGFP-expression was measured by flow cytometry. The suppression of radiation-induced activation of the NF-κB pathway by KU-55933 and MG-132 was determined in HEK-pNF- κB-d2EGFP/Neo L2 cells after exposure to X-rays (200 kV, LET ~0.3–3 keV/µm, (**D**) and ^36^Ar-ions (95 MeV/n, LET 270 keV/µm, (**E**) cells were preincubated with the inhibitor for 1 h and subsequently irradiated. After 18 h, NF-κB-dependent d2EGFP-expression was measured by flow cytometry. The percentage of d2EGFP(+) cells was normalized to the mock-irradiated control. For ^36^Ar ions, doses (Gy) were calculated from fluences (P/cm^2^) according to Equation (1) (Section 4.5.3.).

To determine whether ATM and the proteasome are involved in NF-κB activation by different ionizing radiation qualities, specific inhibitors were used. First, cytotoxicity of the inhibitors and the inhibitory concentration were determined (Figure 2A). 2 µmol/L MG-132 were sufficient to completely suppress TNF-α induced NF-κB activation (Figure 2B). The ATM inhibitor KU-55933 had no effect on TNF-α triggered NF-κB activation, as it is not involved in the classical pathway (Figure 2C). Therefore, the concentration was determined from the literature (10 µmol/L). Treatment with 2 µmol/L MG-132 or 10 µmol/L KU-55933 completely abolished the X-ray-induced NF-κB activation (Figure 2D). Compared to X-rays in the same dose range, exposure with argon ions resulted in a higher NF-κB activation with a saturation at ~8 Gy (Figure 2E). Additionally, this activation was completely suppressed by KU-55933 and by MG-132 (Figure 2E).

### 2.3. Effect of RelA Knock-Down on NF-κB-Dependent Gene Expression

**Figure 3:**
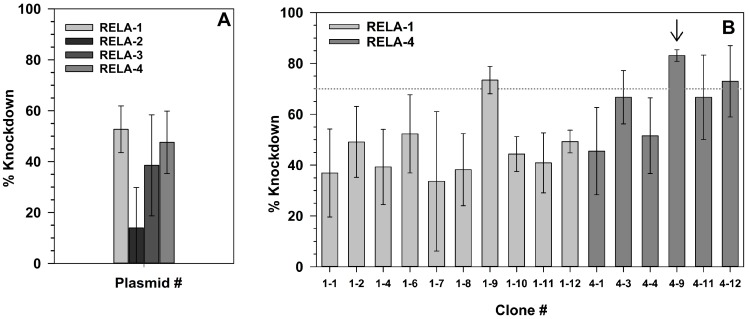
Verification of RelA knock-down on messenger RNA (mRNA) level after transfection of HEK-pNF-κB-d2EGFP/Neo L2 cells with RelA short-hairpin RNA (shRNA) plasmids. Polyclonal hygromycin-resistant cells resulting from transfection with the shRNA plasmids RELA-1 to 4 were grown in petri dishes and total RNA was collected after 3 days (**A**). Clones were grown from the cell population transfected with the shRNA plasmids RELA-1 and 4, as these were most effective in suppressing RelA expression, and the knock-down level of RelA mRNA was determined in these clones (**B**). RelA mRNA expression was determined by real-time RT-qPCR. Means and SD of three independent experiments with each three replicates are shown.

The reporter gene and inhibitor experiments had shown that ATM and the proteasome are involved in ionizing radiation-induced NF-κB activation. For specific downregulation of the NF-κB pathway, the key component RelA was knocked down by stable transfection of four different RelA shRNA plasmids carrying a hygromycin resistance gene. The knock-down level in polyclonal hygromycin-resistant cells resulting from transfection with RELA-1 and -4 plasmids was above 40% (Figure 3A). Clones from these populations were grown, and three clones reached a knock-down level above 70%. The clone with the highest knock-down was selected for further experiments and designated HEK shRNA RelA (Figure 3B, arrow).

As the selected HEK shRNA RelA clone showed a remaining RelA expression of ~17%, the effect of this residual level on basal expression (Table 1) and on TNF-α-induced expression of NF-κB target genes (Table 2) was analyzed.

As expected, RelA mRNA was downregulated. In addition, cyclin D1 and IL-8 expression were reduced in HEK shRNA RelA cells compared to the parental cell line (Table 1). The expression of CCL5, GADD45B and Jun was increased in cells with RelA downregulation.

Incubation with TNF-α induced a large set of NF-κB target genes in HEK cells, with highest expression of TNF, IL8, CXCL1, 2 and 10, and NF-kappa-B inhibitor alpha (NFKBIA) (Table 2). RelA knock-down nearly completely abolishes the TNF-α-induced expression of all these target genes except CCL5. EXO1 which is not a NF-κB target gene is downregulated when RelA is knocked down.

**Figure 4:**
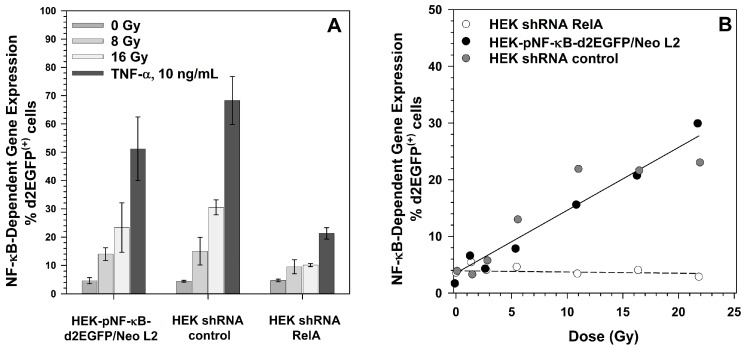
Effect of RelA knock-down on NF-κB activation by X-rays and TNF-α (**A**), and by carbon ions (**B**). HEK-pNF-κB-d2EGFP/Neo L2 cells, cells stably transfected with the shRNA control vector (HEK shRNA control) or the RelA shRNA plasmid (HEK shRNA RelA) were seeded in petri dishes, grown for two days, and exposed to X-rays (200 kV, LET ~0.3–3 keV/µm), incubated with 10 ng/mL TNF-α (**A**) or irradiated with ^13^C-ions (75 MeV/n, LET 34 keV/µm). 18 h after exposure, cells were harvested by trypsination, fixed with 3.5% formaldehyde and the percentage of d2EGFP^(+)^ cells was determined by flow cytometry.

Furthermore, NF-κB-dependent reporter gene expression was determined. The basal level of d2EGFP expression was unchanged compared to the parental cell lines and to cells stably transfected with a shRNA control plasmid with a random sequence (Figure 4A). TNF-α- and X-ray-induced d2EGFP expression were significantly reduced in HEK shRNA RelA cells compared to the parental cell line as well as to the shRNA control cell line (*p* ≤ 0.05, Figure 4A). The reactions of the parental cell line and the shRNA control cell line were not significantly different in no case based on a *p* = 2α level of <0.05. Carbon ion induced d2EGFP expression was completely abolished (Figure 4B).

### 2.4. Growth of RelA Knock-Down Cells

**Figure 5:**
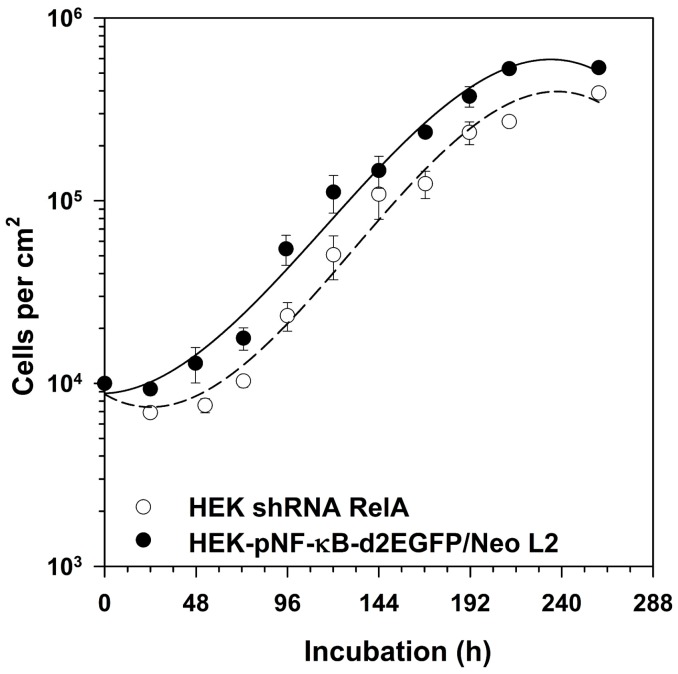
Growth kinetics of HEK shRNA RelA cells compared to the original cell line. 10^4^ cells/cm^2^ HEK-pNF-κB-d2EGFP/Neo L2 cells and cells stably transfected with the RelA shRNA plasmid (HEK shRNA RelA) were seeded in petri dishes. On a daily base, cells were harvested by trypsination and counted in a counting chamber. The graph shows means and standard errors of three independent experiments.

In order to determine whether RelA knock-down affects basic cellular functions such as growth, cell numbers were counted during a growth period of 10 days. HEK shRNA RelA cells showed a prolonged lag phase compared to HEK-pNF-κB-d2EGFP/Neo clone L2 cells (Figure 5). Once proliferation starts, both cell lines grow with the same velocity.

### 2.5. Survival of RelA Knock-Down Cells After X-ray and after Heavy Ion Exposure

**Figure 6:**
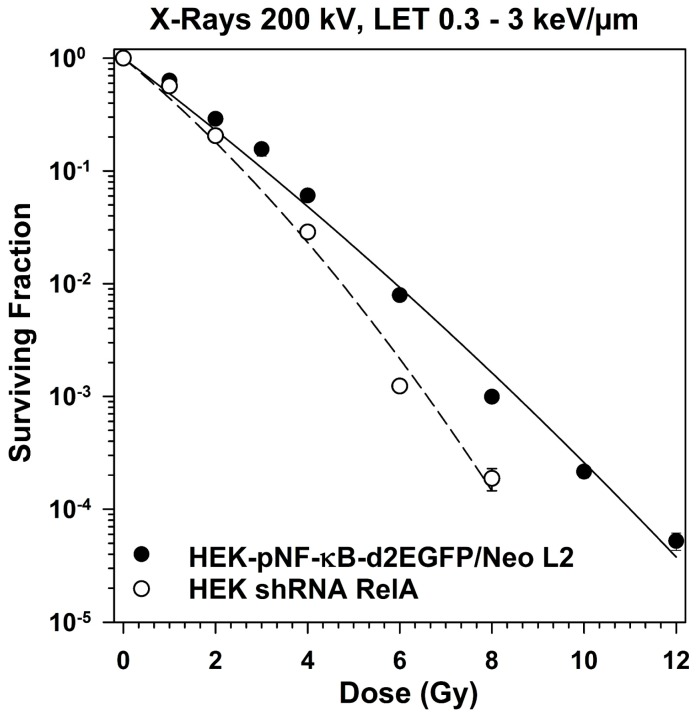
Clonogenic survival of HEK cells with RelA knock-down compared to the parental cells after X-irradiation (200 kV). HEK-pNF-κB-d2EGFP/Neo L2 and HEK shRNA RelA cells were irradiated, incubated and colonies were fixed after 14 to 21 days (means ± SE of 7–13 independent experiments with six replicates each).

The survival curves after exposure of HEK-pNF-κB-d2EGFP/Neo clone L2 cells and HEK shRNA RelA cells were of curvilinear shape (Figure 6). The curve of the RelA knock-down cells is significantly steeper, indicating a higher radiosensitivity. The D_0_ indicating the dose necessary to reduce survival of HEK cells to 37% is 1.12 Gy for the parental cell line compared to 0.82 Gy for the RelA knock-down cells (Table 3).

**Figure 7:**
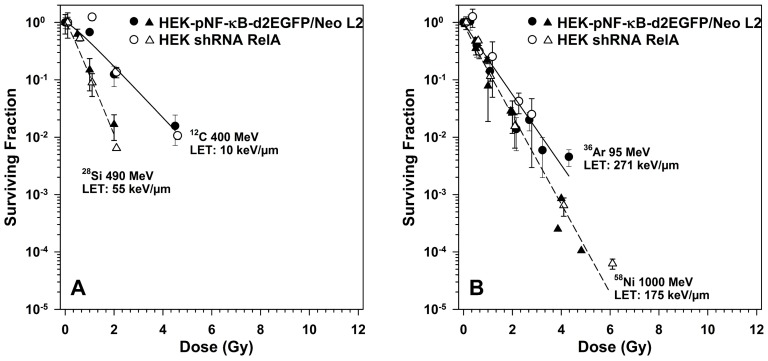
Clonogenic survival of HEK cells with RelA knock-down compared to the parental cells after exposure to heavy ions of diffent LET (**A**), linear energy transfer (LET) < 100 keV/µm, (**B**) LET > 100 keV/µm). HEK-pNF-κB-d2EGFP/Neo L2 and HEK shRNA RelA cells were irradiated, incubated and colonies were fixed after 14–21 days (means ± SE of 1–2 independent experiments with each six replicates).

High-LET radiation exposure of HEK cells results in purely exponential survival curves (Figure 7). Based on energy dose, heavy ions with an LET of 55 keV/µm are most efficient in cell killing (Figure 7A), while radiation qualities with an LET above or below this range are less efficient in cell killing (Figure 7A,B). The D_0_ first decreases to 0.47 Gy for silicon ions, then increases with increasing LET to 0.72 Gy for argon ions (Table 3).

### 2.6. Induction of NF-κB Target Gene Expression by Exposure to Different Radiation Qualities

As NF-κB was weakly activated by X-rays and activated by heavy ions to a higher extent, dependent on LET, but only for X-rays, a reduction of survival in case of RelA downregulation was observed, the expression of 88 NF-κB target genes was profiled 6 h after exposure to X-rays and heavy ions (Table 4).

The expression of chemokines and cytokines was upregulated by low- and high-LET radiation at higher doses. Exposure to 0.5 Gy X-rays or Ti ions did not significantly up-regulate the expression of the investigated NF-κB target genes. The chemokine and cytokine expression was in general higher after exposure to 4 Gy Ti ions compared to 4 Gy X-rays. The highest expression levels were reached for CXCL10 and TNF.

Adhesion molecules such as VCAM-1 and a costimulatory factor (CD83) were also induced. Components of the NF-κB pathway such as NFKB2 and the inhibitor NFKBIA were upregulated by the two radiation qualities.

RelA knock-down inhibited the ionizing radiation-induced NF-κB target gene expression except for TNFRSF1B.

### 2.7. Secretion of TNF-α and IL-8 after X-Irradiation

The secretion of the cytokines GM-CSF, IFN-γ, IL-1β, IL-2, IL-4, IL-5, IL-6, IL-8, IL-10, IL-12(p70) and TNF-α was determined using a multiplex ELISA. Only IL-8 and TNF-α reached values above the quantification limit of the assay.

**Figure 8:**
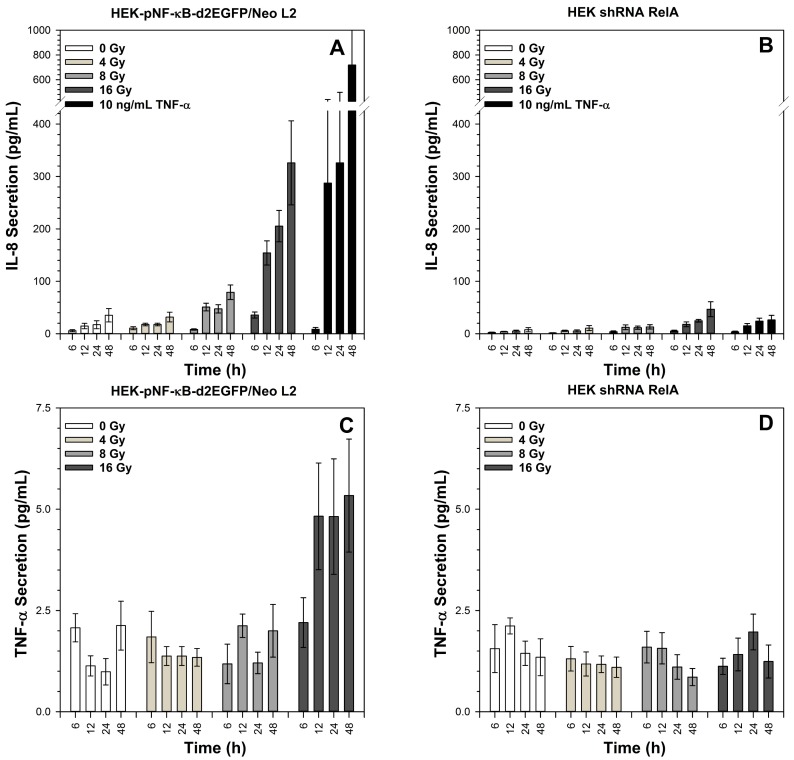
Kinetics of IL-8 and TNF-α secretion of HEK-pNF-κB-d2EGFP/Neo L2 (**A**,**C**) and HEK shRNA RelA cells (**B**,**D**) after X-irradiation. The supernatants were collected at the indicated time points. The TNF-α and IL-8 content of the supernatants was determined by means of a multiplex ELISA. Mean and standard error of three independent experiments with duplicate cytokine determination are shown.

Incubation with TNF-α resulted in strong secretion of IL-8 by HEK-pNF-κB-d2EGFP/Neo L2 cells within 12 h, and the IL-8 concentration in the cell culture supernatants further increased within the next 36 h (Figure 8A). High doses of X-rays (8 and 16 Gy) are required to induce significant IL-8 secretion 12 h after irradiation, and this secretion is further enhanced at later time points (24 h and 48 h). In HEK shRNA RelA cells, the level of IL-8 secretion in mock-irradiated cells (0 Gy) is lower compared to HEK-pNF-κB-d2EGFP/Neo L2 cells, and only the highest X-ray dose (16 Gy) and incubation with TNF-α provoke a very slight time-dependent increase in IL-8 secretion (Figure 8B).

TNF-α secretion increased 12 h after exposure of HEK-pNF-κB-d2EGFP/Neo L2 cells to 16 Gy X-rays and remained elevated during the investigated time period without further significant increase (Figure 8C). The total amount was much lower compared to IL-8. For HEK shRNA RelA cells, TNF-α secretion remained within background levels for all applied doses and incubation periods (Figure 8D). TNF-α secretion induced by incubation with TNF-α is not shown, as the added TNF-α was present in the supernatants and disguised the TNF-α secretion of the cells.

## 3. Discussion

In this study, we show that knock-down of the NF-κB subunit RelA results in sensitization of HEK cells towards low-LET X-rays, but not towards heavy ions in a LET range of 10 to 272 keV/µm. It was previously demonstrated that heavy ions in the LET range of 91 to 272 keV/µm strongly activate NF-κB [20]. The NF-κB activation by low- and high-LET ionizing radiation was shown to be ATM dependent. Furthermore, IκB-degradation by the proteasome is essential for ionizing radiation-induced NF-κB-activation.

RelA knock-down also influenced cell proliferation, resulting in a prolonged lag phase, but it did not impact the proliferation rate once the exponential growth phase was reached. Without NF-κB-activation by TNF-α or ionizing radiation, the reduced RelA levels in the knock-down cells changed the expression of only a few NF-κB target genes (CCND1, CXCL8, JUN, TNF). After incubation with TNF-α or irradiation, the upregulation of CXCL1, 2, 8 and 10, NFKBIA and TNF was strongly reduced in RelA knock-down cells.

### 3.1. Activation of the NF-κB Pathway by Different Radiation Qualities

Our first study examining the effects of heavy ions on the NF-κB pathway revealed that high-LET argon ions (272 keV/µm) activated the sequence of all events in the NF-κB pathway from DNA binding via transcription to protein translation and maturation [18]. The study of the LET dependence of this cellular response to heavy ion exposure disclosed that indeed the maximal potency to activate NF-κB dependent d2EGFP gene expression in HEK cells occurs at 272 keV/µm (argon ions) have [20]. In accordance with the results presented here, other authors using a DNA binding assay report nuclear translocation NF-κB after exposure of normal human monocytes (MM6 cells) after exposure to 0.7 Gy of ^56^Fe ions [29]. Activation of NF-κB was mediated through phosphorylation of IκBα via subsequent proteasomal degradation. The study on iron ion effects on monocytes revealed only binding of NF-κB to its consensus sequence of 5′-GGGGACTTTCC-3′, transcriptional activation itself was not shown.

Also in vivo, NF-κB activation was observed in the heart and bone marrow of mice one week, one month and six months after exposure to 300 MeV/n ^28^Si ions (LET 77 keV/µm) [30]. This clearly indicates that high-LET ion exposure induces rapid and persistent NF-κB activation in vitro and in vivo.

### 3.2. NF-κB Activation by Ionizing Radiation Is ATM Dependent

KU-55933 is specific for ATM with respect to inhibition of other related kinases such as DNA-dependent protein kinase (DNA-PK) or phosphatidylinositol 3′-kinase (PI3K) which required a ~200 times higher concentration for their inhibition [31]. Results showed that while it was not possible to suppress TNF-α-induced NF-κB activation, KU-55933 suppressed ionizing radiation-induced NF-κB activity completely. Increasing doses of X-rays and ^36^Ar ions were used to induce NF-κB, showing its dose and LET dependency, as the percentage of d2EGFP^(+)^ cells increased with increasing doses and ^36^Ar ions had a higher impact on d2EGFP-dependent NF-κB expression than X-rays.

These results support the assumption that ionizing radiation, including high-LET heavy ions, activates the genotoxic stress induced NF-κB pathway and proves that NF-κB activation induced by ionizing radiation is strictly ATM dependent. ATM foci form quickly in the nuclei of human cells after exposure to carbon or iron ions and are believed to facilitate or initiate DNA double strand break (DSB) repair by Non-Homologous Endjoining (NHEJ) and Homologous Recombination (HR) [32,33]. ATM was shown to be activated in human cells to a higher extent by carbon ions (LET 290 keV/µm) than by low-LET γ-rays [34]. This stronger ATM phosphorylation after heavy ion exposure might be important for the strong NF-κB activation that was observed in the LET range of 90–300 keV/µm.

In contrast, TNF-α induced NF-κB activation was not impaired by KU-55933, as ATM is not involved in the classical pathway, which is activated by TNF-α.

### 3.3. IκB Degradation by the Proteasome Is Essential for TNF-α and Ionizing Radiation-Induced NF-κB Activity

Within non-cytotoxic concentrations (viability above 80%), MG-132 suppressed TNF-α- and ionizing radiation-induced NF-κB activity, showing that IκB degradation by the proteasome is an essential step in both, the genotoxic stress and the TNF-α-induced pathway. However, it has to be considered, that disturbing proteasome functions with MG-132 leads to the loss of regulation of essential functions within the cell and these chemical inhibitors are not NF-κB specific.

### 3.4. Role of RelA in Cell Proliferation

Downregulation of RelA prolonged the lag phase. Furthermore, cells required a longer recovery period after freezing and thawing, indicating a role of RelA in cell proliferation. This is supported by the observation that transfection of NF-κB/RelA antisense oligodeoxynucleotide reduces the proliferation of SGC-7901 gastric cancer cells [35] and that siRNA-mediated RelA knock-down decreased the proliferation ability of human esophageal squamous cell cancer (ESCC) EC9706 cells [36]. In tumor cells, the inhibiting effect of RelA knock-down seems to be much more pronounced compared to HEK cells. This might be explained by a constitutive NF-κB activation in many tumors, e.g., some gliomas [37] and hepatomas [38], which is then suppressed. In breast cancer cells, transfection of RelA shRNA reduced proliferation [39].

The reduced expression of cyclin D1 (−3.0) is a possible explanation for the effects of RelA knock-down on proliferation, as RelA and JunD were shown to cooperate to activate the proximal κB site of the cyclin D1 promoter in presence of serum [40]. Cyclin D1 regulates the cyclin-dependent kinases CDK4 or CDK6, the activity of which is required for cell cycle G1/S transition. This connection of NF-κB, cyclin D1 and proliferation was also found in neural stem or progenitor cells (NSCs, NPCs) [41] and fibroblasts [42]. Upregulation of the activated protein 1 (AP-1) family member Jun (5.0) in HEK shRNA RelA cells might be a regulatory mechanism to compensate for the RelA loss, as c-Jun affects cell proliferation, migration and invasion and, like NF-κB, actively participates in tumorigenesis [43].

### 3.5. Role of RelA in Clonogenic Survival after Exposure to Different Radiation Qualities

As activation of the NF-κB pathway is supposed to play a role in the negative regulation of apoptosis, survival of cells with residual DNA damage might thus be favored after irradiation. Even if this happens only in rare cases after ionizing radiation exposure, it might result in initiation or promotion of cancer from such wise affected cells. Cellular responses which may be related to better survival of exposed cells would contribute considerably to astronauts’ radiation risk, especially at low doses when transient induction of genes involved in maintaining DNA fidelity and in modulating cell cycle progression and cell death would occur [44]. As heavy ion exposure strongly activated NF-κB, we examined whether NF-κB gives HEK cells a survival advantage after heavy ion exposure. For this purpose, expression of the NF-κB subunit RelA was downregulated by stable transfection of RelA shRNA.

The colony forming ability test showed that HEK cells were more sensitive to X-irradiation when RelA was downregulated. Such sensitization to X-rays was also observed with chemical NF-κB inhibitors, e.g., the sesquiterpene lactone parthenolide, an IκB kinase (IKK) inhibitor, inhibited constitutive and radiation-induced NF-κB binding activity in p53 null PC-3 cells and enhanced their X-ray sensitivity by a dose modification factor of 1.7 [45]. RelA knock-out murine embryonic fibroblasts (MEF p65^−/−^) were 1.3-fold more sensitive towards X-rays than the p65^+/+^ cells [46]. In many cancer cell types, NF-κB inhibition sensitized the cells to ionizing radiation [47,48,49,50].

In these MEF p65^−/−^ cells, the killing effect of UVB radiation was lower compared to MEF wildtype cells (p65^+/+^) [51]. Therefore, in contrast to ionizing radiation, loss of RelA conferred a strong resistance to UVB-induced cell death which was accompanied by reduced Gadd45 gene expression [51].

In contrast to the sensitization to X-rays by RelA knock-down, the sensitivity to heavy ion exposure was equal or only slightly different for cells with and without RelA over a wide range of LET (10–271 keV/µm). This indicates that RelA and heavy ion induced NF-κB activation does not procure a survival advantage for the exposed cells. This might be explained by other pro-death signals of the heavy ion exposure. Interestingly, in human hematopoietic stem and progenitor cells (HSPC) and peripheral blood lymphocytes (PBL), inhibition of NF-κB activation by expression of a super-repressor variant of IκBα (IκBα-SR) altered DNA double-strand-break repair [52]. IκBα-SR expression reduced homologous DSB repair in PBL by 33% [52]. NHEJ of DNA DSBs was compromised in HSPC and PBL (reduction to 37% and 52%, respectively) [52]. These measurements were performed on enzymatically induced DNA DSBs, results for low- or even high-LET radiation-induced DSBs are not yet available. Nevertheless, reduced DNA DSB repair in case of NF-κB repression or down-regulation might play a role for survival after ionizing radiation exposure. Delayed or incomplete repair might override other NF-κB induced survival signals. Another explanation for the missing effect of RelA knockdown might be the high-LET which is in the range of the highest observed biological efficiency. The cell killing effect in the LET range of 100–200 keV/µm is already very high, so that disturbed cellular signaling pathways might not be able to further increase cell killing. This was observed for the pro-apoptotic p53 in some cell systems: apoptosis was independent of the p53 status [53,54].

Comparing ionizing radiation and chemotherapeutics such as the topoisomerase II inhibitor doxorubicin, knock-down of RelA by siRNA increased the killing effect of doxorubicin in HepG2 cells [38]. The authors of this publication explain the increased killing of hepatoma cells by lower NF-κB-mediated expression of multi-drug resistance (MDR1) gene [38]. A mechanism based on MDR1 is not likely to influence survival after ionizing radiation. In nude mice bearing HeLa cervical cancer xenografts, downregulation of p65 via photothermal transfection of p65 siRNA increased irinotecan-induced tumor apoptosis [55].

Furthermore, the NF-κB target gene expression profile might be different for low- and high-LET radiation exposure and might give an explanation for the different role in the cellular radiation response. Therefore, the expression of 88 target genes was determined after X-ray and heavy ion exposure. Genes that are regulated more than threefold and at the same time are controlled by RelA are discussed below.

### 3.6. Role of RelA in the Expression of NF-κB Target Genes after Exposure to Different Radiation Qualities

**Figure 9:**
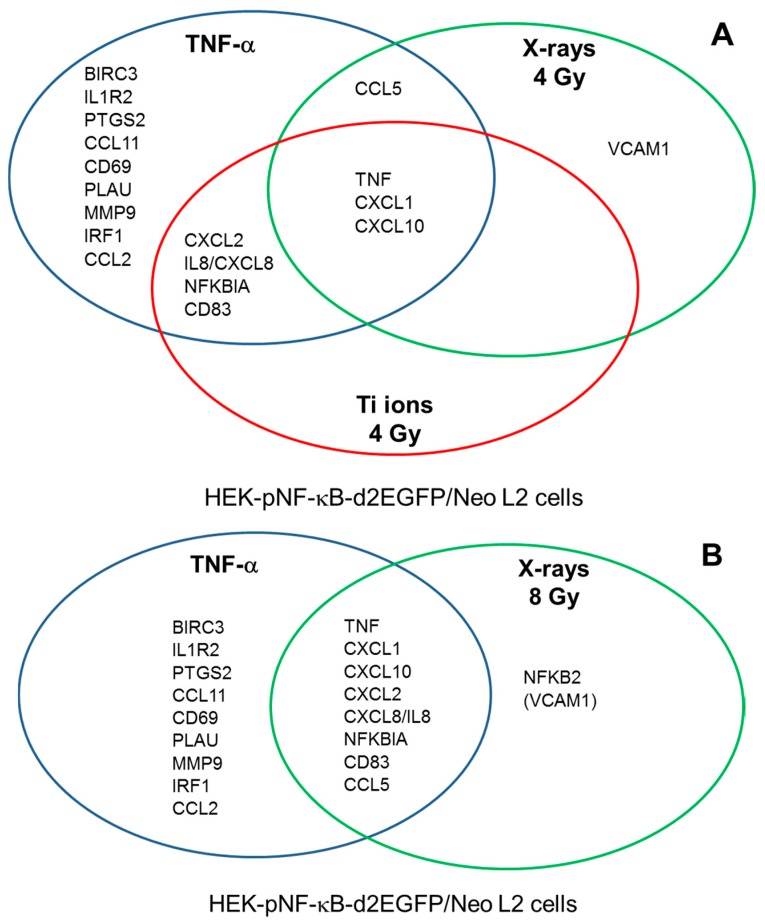
Diagram showing the expression of NF-κB target genes by HEK cells 6 h after exposure to 10 ng/mL TNF-α, 4 Gy X-rays or 4 Gy Ti ions (**A**) and after exposure to 10 ng/mL TNF-α or 8 Gy X-rays (**B**) VCAM-1 is in parentheses as its expression is just below the threshold of threefold induction.

NF-κB target genes that are induced after irradiation affect intercellular communication and many of them are proinflammatory. e.g., TNF-α activates inflammation by inducing a proinflammatory cytokine cascade, mediated through expression of IL8 [56]. Incubation with TNF-α induces the expression of a larger subset of genes than low- or high-LET irradiation at a dose of 4 Gy (Figure 9A). TNF, CXCL1 and −10 are upregulated by TNF-α, 4 Gy X-rays and 4 Gy Ti ions. A higher X-ray dose (8 Gy) increased the overlap in the subset of induced NF-κB target genes (Figure 9B). The gene subsets show overlap with those determined in osteosarcoma cells [57].

The overlapping gene expression in HEK-pNF-κB- d2EGFP/Neo L2 cells after TNF-α and radiation treatment was not observed in RelA knock-down cells, confirming the role of the NF-κB subunit RelA in the expression of these genes.

After TNF-α treatment HEK-pNF-κB-d2EGFP/Neo L2 cells but not in HEK shRNA RelA cells showed a clear upregulation of C-X-C motif (CXC, C stands for cysteine, X for any other amino acid) chemokine genes, including CXCL1, CXCL10, CXCL2 (MIP-2) and CXCL8 (IL8). The CXC chemokines belong to one group of small cytokines, which, when they are are secreted by infected cells act as chemoattractants inducing chemotaxis or migration especially of neutrophils, which are the first responders during inflammation to migrate towards site of inflammation. Additionally, in wound healing the increased chemokine concentration of damaged cells results in attracting cells to site of damage. Loss of chemokine induction is a serious impairment for the organism as it leads to dysfunctional cell migration and wound healing deficiency. The essential role of TNF in the early induction of chemokines and subsequent recruitment of leukocytes have been shown using models of mycobacterial infection [58] as induction of chemokines and cellular recruitment were delayed in TNF^−/−^ cells. Animal studies show that CXCL2 was upregulated in murine tumors after exposure to carbon ions (290 MeV/n, LET 50 keV/µm) [59].

As expected, TNF-α treatment induced high upregulation (60.0) of TNF, as TNF-α, after being released as a response to immunological stimuli, acts as a positive autocrine feedback signal to augment NF-κB activation [60]. In the knock-down cell line, no upregulation of TNF was observed, as NF-κB is a key transcription factor involved in the synthesis of TNF-α [61].

Upregulation of NFKBIA (encoding IκBα) in the original cell line was expected as well, as in addition to increasing the transcription of cytokines and adhesion proteins, NF-κB also increases the transcription of the inhibitor IκB, thus leading to its own inactivation and subsequent termination of the response [62]. Other studies showed that NFKBIA was also upregulated in six human malignant melanoma cell lines after carbon ion exposure (290 MeV/n, LET 50 keV/µm) [63].

Only three of the genes upregulated in HEK-pNF-κB-d2EGFP/Neo L2 cells were induced by TNF-α in the knock-down cell line: CCL5, TNF and TNFRSF1B. For the latter two genes, the induction factor was much lower compared to the RelA proficient cell line. Instead, downregulation of one NF-κB target gene was observed, EXO1. Exonuclease 1 (EXO1) is involved in DNA damage signaling [64].

The target gene array, investigating 88 genes after X- and ^48^Ti ion irradiation, showed that NF-κB-dependent gene expression does not only depend on dose, but also on LET, complementing our recently published study [26] and studies with *Caenorhabitidis elegans* [65]. The extent of gene expression increases with increasing doses. Further, the degree of regulation is stronger after 4 Gy of ^48^Ti ion exposure than after X-irradiation of the same dose.

In HEK-pNF-κB-d2EGFP/Neo L2 cells, the set of genes, that has already been shown to be upregulated after TNF-α treatment, was likewise upregulated after exposure to X-rays and heavy ions. It includes CXC chemokines, TNF and NFKBIA which encodes the NF-κB inhibitor IκBα. This shows that expression of these genes is activated not only by the classical NF-κB pathway, but also the genotoxic stress induced, independent of the radiation quality.

In this work, both low- and high-LET radiation exposure resulted in upregulation of TNF, and with higher LET, the upregulation was stronger. Interestingly, in the tibial marrow of Fe ion (600 MeV/n, LET 174 keV/µm) irradiated mice, no significant increase in TNF expression was found three days after exposure to 2 Gy, and only a slight increase after 2 Gy γ-irradiation which remained elevated through day three after irradiation [66].

Especially the CXC chemokines are strongly upregulated, in case of CXCL10 up to 14.6 fold after irradiation with 8 Gy X-rays. None of the genes that are upregulated at higher doses in the original cell line exceeds the threshold of 3 after irradiation with 0.5 Gy of both, X-rays and ^48^Ti ions. Though, after 4 Gy of X-rays, CXC chemokines reach expression values of 2.3 to 4.7 fold. For the same dose, these genes are expressed up to 7.6 fold after ^48^Ti ion irradiation.

CCL5 has chemotactic activity for monocytes and basophils and binds to chemokine receptors CCR2 and CCR4. It was upregulated by high doses of X-rays. Such an upregulation was also observed in vivo in the bone marrow of C57BL/6J mice exposed to 2 Gy γ-rays and is suggested to promote ostoclastogenesis [66].

Apart from the genes expressed after both, TNF-α treatment and irradiation, additional genes are upregulated only upon X-irradiation, not after heavy ion exposure, proving the relation between radiation quality or LET and gene expression. These genes include CCL5, NFKB2, and VCAM1.

NFKB2 codes for the NF-κB subunit p100, which is involved in the non-canonical NF-κB pathway. It is processed to p52. This element of the non-canonical pathway seems to be upregulated only by X-rays and not by heavy ions. The RelB:p52 dimer was shown to confer radio-resistance to prostate cancer cells by upregulating the expression of the antioxidant enzyme manganese superoxide dismutase (MnSOD) [67,68,69,70]. An upregulation of RelB expression 8 h after X-ray exposure (0.1, 1 Gy) was also observed in a three-dimensional skin tissue model [71].

The different gene expression profile after ^48^Ti ion irradiation indicates distinct responses, depending on radiation quality, eventually including different repair mechanisms, as indicated in the survival curves in this work. These genes are expressed after X-irradiation, but not after ^48^Ti ion exposure. The missing expression of the protecting RelB gene after heavy ion exposure might be another explanation why RelA knock-down results in higher X-ray sensitivity, but not in higher heavy ion exposure sensitivity: Its protecting effect ceases to exist in HEK shRNA RelA cells after X-irradiation, but it is not induced after heavy exposure even in case of normal RelA levels.

CD83 expression was activated by 4 Gy Ti ions, TNF-α and 8 Gy X-rays. It is one of the central regulatory molecules in immune functions with anti-tumor effects [72]. It acts as a co-stimulator during activation of T-cells by antigen-presenting cells and is therefore essential for the induction of the adaptive immune response. NF-κB regulates inducible CD83 gene expression in activated T lymphocytes during an adaptive immune response [73]. Increased expression of this costimulatory molecule indicates that exposure to ionizing radiation does not only induce inflammation as a prominent innate immune response via proinflammatory cytokines such as TNF-α and via chemokines, but also induces selected steps towards activation of adaptive immunity.

In absence of RelA, only TNFRSF1B is upregulated after X-ray exposure. TNFRSF1B encodes for the TNF receptor TNF-R2. It was shown that TNFRSF1B receptor signaling resulted in the activation of anti-apoptotic survival proteins [74]. At the same time, TNFRSF1B promotes TNF-α induced apoptosis [75]. Baseline plasma TNFRSF1B, a marker of inflammation and a soluble TNF antagonist, was significantly associated with an increased risk of human colorectal cancer [76]. TNF-α bound to TNFRSF1B recruits intracellular adaptor proteins to activate multiple signal transduction pathways including NF-κB [77]. The activation of NF-κB in intestinal epithelial cells by TNF-α through TNFRSF1B receptor signaling has been previously linked to carcinogenesis. Silencing TNFRSF1B increased apoptosis [74]. However, NF-κB interferes with apoptotic signals at various levels. The best example is found in the TNF-R1 pathway [78]. It is therefore not certain whether increased apoptosis results from pro-apoptotic TNFRSF1A signaling. An upregulation of TNFRSF1B expression 8 h after X-ray exposure (0.1, 1 Gy) was also observed in a three-dimensional skin tissue model [71].

Overall, results revealed the outstanding role of NF-κB and its subunit RelA in cytokine expression after exposure to low- and high-LET radiation and therefore in the “sterile inflammation” after radiation exposure [57]. In contrast to other studies with cancer cells, no radiation-induced upregulation of antiapoptotic genes above the threefold threshold was found. BIRC3 encoding cIAP2 was upregulated 2.0 and 2.5 times after exposure to 4 and 8 Gy X-rays, respectively. In differentiated thyroid cancer (DTC) cells, incubation with (131)I-induced NF-κB activation, and knock-down of p65 by siRNA transfection increased (131)I-induced cell killing [79]. This effect was explained by lower expression of the antiapoptotic NF-κB target genes XIAP, cIAP1, and Bcl-xL when p65 expression was downregulated by siRNA [79].

### 3.7. Role of RelA in IL-8 and TNF-α Production for Survival after Exposure to Different Radiation Qualities

IL8 (Interleukin 8, CXCL8) was severely downregulated (−8.1) in HEK shRNA RelA cells. In presence of RelA, IL-8 secretion was strongly induced by TNF-α and high X-ray doses. This cytokine is part of the CXC chemokine family and acts as proinflammatory mediator, further activating proinflammatory cytokines in a positive feedback loop. It attracts and activates neutrophils [80], causing them to migrate towards the site of infection. It was shown for several cell types, that the NF-κB binding site is essential for transcription of IL-8 [81,82]. Interleukin 8 secretion was also increased in response to oxidative stress, mediated by TNF-α, which further induces RelA-binding to the binding site of the IL-8 promoter [56]. In HTori-3 cells, a human thyroid epithelial cell line, exposed to either a non-toxic radiation dose (0.1 Gy) or a slightly toxic dose (0.2 Gy) from iron ions (1 GeV/n), IL-8 expression was altered [83]. In organotypic cultures of oral mucosa irradiated with X-rays or carbon ions, both radiation qualities increased the release of IL-8, with a saturation at 4 Gy for early time points, and higher early increases (4 h) after carbon ion exposure in the spread-out Bragg peak (SOBP, track-averaged LET ~50 keV/µm) [84,85]. The IL-8 release remained increased for two days after carbon ion exposure (2, 4, 10 Gy) [84].

TNF-α secretion was increased after higher doses of X-rays (16 Gy), while its mRNA level was enhanced already at a dose of 4 Gy.

### 3.8. Implications for Countermeasure Development

Although caution should be taken to directly translate in vitro data to a clinical setting and the doses used here are quite high, the study suggests that due to NF-κB activation by heavy ion exposure astronauts would be prone to “sterile inflammation” in relevant target organs. Indeed, Parihar et al. found increased numbers of activated microglia in the perirhinal cortex of rodents exposed to low doses of helium and other space-relevant accelerated ions, indicating neuroinflammation [86,87].

For ensuring astronauts’ health, countermeasures have to be developed which could rely not necessarily on therapeutically applied drugs but on natural inhibitors of the proteasome or other regulating factors in the NF-κB pathway as dietary supplements. The proteasome inhibitor MG132 which was used in this study is not such a candidate as it inhibits multiple proteases in addition to the proteasome. Furthermore, at least in tumor cells, treatment with MG-132 prior to irradiation lead to radiosensitization [88], which is an effect exploited for tumor therapy with the proteasome inhibitor bortezomib [89].

Possible candidates for protection against chronic inflammation could be members of the triterpenoid family, which inhibit the NF-κB pathway at various sites [90,91]. Additionally, in our earlier studies, capsaicin, an active component of the red pepper (*Capsicum*), partially inhibited NF-κB activation in HEK cells in a non-toxic concentration range [92]. However, NF-κB is a central element of various physiological functions and its inhibition might be associated with various side effects. Therefore, evaluation of its target genes that are upregulated by radiation exposure might lead to more specific toeholds to mitigate radiation-induced inflammation.

For high-dose exposure that could occur during a large solar particle event, activation of the NF-κB pathway might increase the survival of, e.g., bone marrow stem cells and intestinal epithelial cells and thereby ameliorate the acute radiation syndrome. For this purpose, Toll-like receptor 5 (TLR5) agonists were developed based on bacterial flagellin and successfully tested in animal models [93].

### 3.9. Conclusions

RelA is essential for cytokine and chemokine expression after exposure to both, low- and high-LET radiation and it thereby shapes the sterile inflammatory response, which comprises a smaller NF-κB target gene subset compared to TNF-α treatment. ATM and the proteasome are essential for NF-κB activation by different radiation qualities. NF-κB confers a survival advantage after X-ray, but not after heavy ion exposure.

## 4. Materials and Methods

### 4.1. Transformation and Preparation of Plasmid DNA

For plasmid propagation, 100 μL of competent *E. coli* DH5α-cells (Stratagene; Agilent Technologies, Karlsbrunn, Germany) were transformed with 2 μL of each SureSilencing™ shRNA Plasmid (~30–50 ng/μL) according to the manufacturer’s protocol. The transformed bacteria were plated on selective LB-agar plates containing ampicillin (Biochrom GmbH, Berlin, Germany) with a final concentration of 50 μg/mL for selection and incubated at 37 °C over night. For plasmid preparation, single colonies were cultured for 16 h in 100 mL of LB-medium containing ampicillin under permanent shaking. For preparation of plasmid DNA the Plasmid Maxi Kit (Qiagen, Hilden, Germany) was used following the instructions of the manufacturer. For quality control, the optical density at 260 nm and 280 nm was determined in a spectrophotometer (Nanodrop 2000c, Thermo Scientific, Langenselbold, Germany). Only preparations with an OD260/OD280 of more than 1.8 were used for the transfection of human cells.

### 4.2. Cell Culture

The cell line HEK 293 was established by Graham et al. (1977) from human embryonic kidney cells immortalized by transfection with sheared fragments of adenovirus type 5 DNA [94]. The cells contain only the stably integrated region (E1a and E1b genes) of the human adenovirus genome and do not produce viral particles. Cells were obtained from the American Type Culture Collection (ATCC CRL-1573; now: LGC Standards, Wesel, Germany). Cloning of the plasmid pNF-κB-d2EGFP/Neo, stable transfection and selection of an appropriate cell clone (HEK-pNF-κB-d2EGFP/Neo L2) was already described [27]. HEK-pNF-κB-d2EGFP/Neo L2 and untransfected HEK cells have comparable radiosensitivity to X-rays and to accelerated carbon ions and show no difference in proliferation [19]. Cells were maintained in α-medium (Biochrom KG, Berlin, Germany) containing 0.6 mg/mL G418 and 10% fetal bovine serum (FBS) at standard conditions (37 °C, 95% air and 5% CO_2_ atmosphere). Cells were passaged every week and seeded in a cell density of 3 × 10^4^ cells/cm^2^ in 80 cm^2^ cell culture flasks (Nunc, Novodirect, Kehl, Germany). Medium was exchanged after four days. Cell culture vessels were coated with poly-d-lysine (10 µg/cm^2^, Sigma-Aldrich Chemie, Steinheim, Germany, in sterile deionized water) to allow a stronger binding of cells to the substrate. Therefore, the culture vessels were coated with poly-d-lysine for 15 min at room temperature and subsequently washed three times with sterile deionized water.

### 4.3. Stable Transfection

For knock-down of the NF-κB subunit RelA, HEK-pNF-κB-d2EGFP/Neo L2 were stably transfected with the SureSilencing™ shRNA plasmids RELA-1 to 4 and the control plasmid from SABiosciences (Frederick, MD, USA). Four of these plasmids encoded different targeting sequences of RelA mRNA, while the fifths plasmid was a negative control vector containing a scrambled artificial sequence which did not match any human, mouse or rat gene. HEK-pNF-κB-d2EGFP/Neo L2 cells were seeded in poly-d-lysine-coated 6-well-plates (Nunc). Transfection was conducted at a cell density of ~50%. Therefore, 2 μg of DNA of each vector were mixed with the transfection reagent FuGENE 6^®^ (Roche, Mannheim, Germany) and serum-free α-medium according to the protocol. After three days of incubation, cells were transferred into ∅ 6 cm culture dishes (Nunc) containing α-medium with 400 μg/mL hygromycin (US Biological, Salem, MA, USA). After approximately 10 days, cells were seeded for RNA isolation (Section 4.9.1) to measure the knock-down level of RelA in the mixed population by real-time quantitative Reverse Transcriptase Polymerase Chain Reaction (RT-qPCR). The strongest knock-down of RelA expression was achieved with the plasmids RELA-1 and RELA-4 (Figure 4A). Therefore, clones grown from single cells of these two cell populations were grown by seeding 50 cells per microtiter plate containing hygromycin-medium. Cells were incubated for 2–3 weeks until colonies were grown. A single colony was washed with PBS, trypsinized and added to a 25 cm^2^ cell culture flask (Nunc) containing 5 mL of hygromycin-medium. When cells had reached a density of 80–100%, their knock-down level was determined by seeding them in triplicates and proceeding according to 4.10. In clone 4–9, RelA mRNA expression was reduced by 83.1% and it was used for further experiments. The selected clone was named HEK shRNA RelA and is characterized by a RelA knock-down level of 83.1% and a resistance against hygromycin.

### 4.4. Growth Kinetics

To characterize differences in growth behavior between HEK-pNF-κB-d2EGFP/Neo L2 and HEK shRNA RelA, both cell lines were seeded with the same cell number (1 × 10^4^ cells/cm^2^) in poly-d-lysine-coated 6-well plates. The cell number of one well was determined daily until cells reached the stationary growth phase.

### 4.5. Treatment Modalities

For cytokine treatment or irradiation experiments, 3 × 10^4^ cells/cm^2^ were seeded into suitable culture vessels freshly coated with poly-d-lysine (10 µg/cm^2^, Sigma-Aldrich Chemie, Steinheim, Germany) for 15 min followed by washing with phosphate buffered saline (PBS). For exposure to TNF-α, X-rays and heavy ions, cells were seeded into 25 cm^2^ flasks (Iwaki, Dunn Labortechnik GmbH, Asbach, Germany for GANIL, Nunc for GSI and HIMAC). Cells were incubated for two days before treatment.

#### 4.5.1. Cytokine Treatment

Human recombinant TNF-α was obtained from Sigma-Aldrich Chemie. TNF-α (final concentration 10 ng/mL), was dissolved in PBS, and added in serum-containing medium. Cells were harvested for flow cytometry as described in 0 after different time points. Control dishes were solvent-treated.

#### 4.5.2. X-Irradiation

X-irradiation was performed at DLR in Cologne using a Gulmay RS225 X-ray source (Xstrahl, Surrey, UK). Cells were irradiated at room temperature with 200 kV and 15 mA at a dose rate of ~1 Gy/min. To eliminate soft X-rays, a copper filter (thickness 0.5 mm) was used. Cells were irradiated in petri dishes (Ø 3 or 6 cm). Doses up to 16 Gy were used, with an LET of 0.3–3 keV/μm. Control dishes were sham-irradiated. Doses were determined using the UNIDOS^webline^ (PTW, Freiburg, Germany).

#### 4.5.3. Heavy Ion Exposure

Irradiation with accelerated heavy ions was performed at the “Grand Accélérateur National d’Ions Lourds” (GANIL, Caen, France), at the GSI Helmholtzzentrum für Schwerionenforschung GmbH (GSI) in Darmstadt, Germany, or at the Heavy Ion Medical Accelerator in Chiba (HIMAC), Japan. Characteristics of the applied beams including the LET in water are shown in Table 5.

Adherent cells were irradiated in sub-confluent stage and in upright position at room temperature. Therefore, flasks and petri dishes were completely filled with α-medium and closed. Dosimetry was performed by the staff at the accelerator facilities. Dose rates were adjusted to approximately 1 Gy/min. At GANIL, dosimetry was performed by the “Centre de recherches sur les Ions, les Matériaux et la Photonique” (CIMAP)/“Laboratoire d’Accueil en Radiobiologie avec les Ions Accélérés” (LARIA) staff, yielding the fluence in particles/cm^2^ (P/cm^2^) [95]. To convert fluence (F) to the energy dose, the following formula was applied [96]:

Dose[Gy] = 1.6 × 10^−9^ × LET[keV/µm] × F[P/cm^2^] (1)

After irradiation, serum-free α-medium was discarded and cells were further cultivated with fresh medium containing 10% FBS.

### 4.6. Cytotoxicity of Chemical Inhibitors

Cytotoxicity of chemical inhibitors KU-55933 and MG-132 was measured by the MTT assay. Reduction of MTT (3-(4,5-dimethylthiazole-2-yl)-2,5-diphenyltetrazolium bromide) to formazan is correlated to the activity of cellular enzymes and measured colorimetrically. HEK-pNF-κB-d2EGFP/Neo L2 cells were seeded in 96-well plates and incubated with each inhibitor for 20 and 72 hours. NF-κB was activated by adding TNF-α protected from light. 100% DMSO was added to one well to measure the background signal. After removing medium, cells were incubated for 1 h at 37 °C with serum-free α-medium containing 0.1 mg/mL MTT. Subsequently, cells were lysed with DMSO with 10% (*w*/*v*) sodium dodecyl sulfate (SDS) and 0.6% (*v*/*v*) acetic acid while shaking them for 20 min with 30 rpm. Finally, absorbance of formazan was measured with the fluorescence/luminescence microplate reader Lambda Fluoro 320 (MWG Biotech AG, Penzberg, Germany) at 562 nm. Cell survival was calculated as a ratio of formazan-absorbance of treated vs. untreated samples after subtraction of the background signal.

### 4.7. Colony Forming Ability

For investigating cellular survival after exposure to X-rays and heavy ions, cells were seeded and irradiated as already described. Immediately after irradiation adherent cells were trypsinized and re-plated in six petri dishes per dose. The cell density for seeding of irradiated cells was adjusted to compensate for the plating efficiency of the respective cell line and for the anticipated lethal effect of the treatment in order to allow growth of 40–60 colonies per dish.

After 21 days of incubation without medium change the resulting colonies were fixated and stained (1 mg/mL crystal violet in 3% formaldehyde solution, Sigma-Aldrich Chemie) and colonies containing more than 50 cells were counted. The experiments were performed with six dishes per dose and repeated if beam time was available.

All data from the irradiated samples were fitted by least-squares linear regression analysis to lnS = ln(PE_D_/PE_D=0_) versus dose, where the natural logarithm of S is the survival and PE are the plating efficiencies of treated (PE_D_) or untreated cells (PE_D=0_). Differences in the survival curves were statistically verified by a two-sided Student’s *t*-test of the regression lines. A *p* level of 0.05 was considered to reflect significance.

### 4.8. Flow Cytometry

For measurement of d2EGFP, cells were harvested at different time points after radiation or cytokine treatment by trypsinization and fixed with 4.5 mL cold 3.5% formaldehyde in PBS. For flow cytometric analysis, cells were centrifuged and resuspended in PBS. Forward and side scatter and green fluorescence (FL-1) of 20,000 cells from the samples were measured in a FACScan (Becton Dickinson, San Jose, CA, USA) with an argon laser (488 nm) as excitation source. The markers d2EGFP^(−)^ and d2EGFP^(+)^ were set by means of untreated and TNF-α treated cells. The percentage of d2EGFP^(+)^ cells was used as measure of NF-κB-dependent d2EGFP expression.

### 4.9. Gene Expression Analysis

#### 4.9.1. RNA Isolation

Each sample was seeded in triplicates. At a density of ~50% cells were lysed with RLT buffer containing 1% 14.3 mol/L β-mercaptoethanol and transferred into an RNase-free Eppendorf tube with an iced syringe. RNA was isolated using the RNeasy^®^ Mini Kit (Qiagen, Hilden, Germany) according to the manufacturer’s protocol. As RNA was used for cDNA synthesis and subsequent RT-qPCR, the additional DNase treatment step was included to eliminate residual genomic DNA. RNA was eluted in 50 μL RNase-free H_2_O.

RNA concentration and purity were measured photometric (A260/280) with the NanoDrop 2000c Spectrometer (Thermo Scientific, Waltham, MA, USA). Additionally, RNA concentration, RNA integrity (RNA Integrity Number; RIN) and rRNA ratio (28S/18S) were measured by micro-electrophoresis using the RNA 6000 Nano Assay in the Agilent 2100 Bioanalyser (Agilent Technologies, Santa Clara, CA, USA).

For gene expression analysis with the RT2 Profiler™ PCR Arrays (SABiosciences, Washington, DC, USA), further RNA quality control with RT2 RNA QC PCR array (SABiosciences) was performed.

#### 4.9.2. cDNA Synthesis

Quality, integrity and quantity of isolated RNA were analyzed. Subsequently, RNA was transcribed into cDNA using the RT2 First Strand Kit (SABiosciences). A preliminary genomic DNA elimination step was conducted by incubation of 1 µg total RNA with gDNA Elimination Buffer, as recommended in the protocol. To detect possible remaining genomic DNA contamination in isolated RNA samples, one sample lacking reverse transcriptase was used as a negative control. The reaction mix (4 μL BC 3 (5X RT Buffer), 2 μL PC2 (Primer and External Control Mix 3), 2 μL RE3 (RT Enzyme Mix 3), 3 μL ddH_2_O) was incubated at 42 °C for 5 min. The RT reaction mix was then added to the Genomic DNA Elimination Mixture and incubated at 42 °C for 15 min. The reaction was stopped by heating at 95 °C for 5 min. cDNA concentration at this stage was 50 ng/μL. Finally, 91 μL of H_2_O were added to each 20 μL cDNA synthesis reaction to obtain a final cDNA concentration of 9 ng/μL. cDNA samples were stored at −20 °C.

#### 4.9.3. Setting Up RT-qPCR Validation of Knock-Down-Level

The quantitative real-time PCR reaction mix for one PCR tube was composed referring to the RT2 qPCR Primer Assays protocol (SABiosciences) as follows: 12.5 μL RT2 SYBR Green qPCR Master Mix, 8.5 μL ddH_2_O, 3 μL Template cDNA (27 ng) and 1 μL 10 μmol/L PCR primer pair stock.

As a negative control DNA was replaced by H_2_O. The primer pairs SYBR^®^ Green Human RELA and SYBR^®^ Green Human HPRT 1 (RT2 qPCR Primer Assay, SABiosciences) were used for targeting the gene of interest (GOI) RelA and the housekeeping gene (HKG) HPRT (Table 6).

#### 4.9.4. Human RT2 Profiler PCR Array

RT2 Profiler™ PCR Arrays (SABiosciences) were used to profile the expression of 88 genes per sample. Two different arrays were available, with one being customized (PAHS-021), individually composed of genes of interest of several signaling pathways (Human Cell Cycle, PAHS-020A; Human NF-κB Signaling Pathway, PAHS-025A; Human p53 Signaling Pathway, PAHS-027A; Human Apoptosis, PAHS-012A; Human PI3K-AKT Signaling Pathway, PAHS-058A; Human Stress and Toxicity Pathway Finder, PAHS-003A; Human DNA Damage Signaling Pathway, PAHS-029A; Human Oxidative Stress and Antioxidant Defense, PAHS-065A) for an initial screening (Table A2) and one array focusing on genes targeted by NF-κB (human NF-κB target gene array, PAHS-225ZD, Table A1). An overview of all genes included in these arrays is listed in Appendix Tables A1 and A2. Overall, 162 genes, involved in cell cycle control, apoptosis, DNA damage, stress and toxicity and NF-κB signaling were investigated. Each well of these arrays contains primers for the respective gene.

Only cDNA samples, which had been approved by the RT2 RNA QC PCR Array (SA Biosciences) to prevent false positive signals by genomic DNA contamination, were used for further analysis. The RT-qPCR master mix for one 96-well plate was set up according to the RT2 RNA QC PCR Array protocol using 1350 μL RT2 SYBR Green qPCR Master Mix, 1248 μL ddH_2_O and 102 μL template cDNA (9 ng/μL).

Quantitative real-time analysis was performed in the Opticon2 thermocycler starting with an initial denaturation for 10 min at 95 °C, following by 40 cycles (denaturation for 15 s at 95 °C, annealing for 35 s at 55 °C, elongation for 30 s at 72 °C, plate read), a final incubation for 1 min at 95 °C and 2 min at 65 °C, and a melting curve from 65–95 °C with reads every 0.2 °C. The threshold cycle (Ct) was determined in the amplification plots. Results were analyzed according to the algorithm described in the RT2 RNA QC PCR Array protocol. It is based on the ∆∆Ct method:

∆Ct = Ct (target gene) − Ct (reference gene)(2)

∆∆Ct = ∆Ct (sample) − ∆Ct (control)(3)

The relative expression of treated sample versus control was calculated by means of Equation (4):

Rate of gene expression = 2^−∆∆*C*t^ (4)

In a cautious data interpretation, the threshold for significant up- or downregulation was set to −3 or +3, respectively, to avoid misinterpretation of physiological fluctuation of mRNA levels.

### 4.10. Measurement of Cytokine Secretion

To measure the cytokine concentration in the cell culture supernatants (100 µL aliquots, frozen and stored at −80 °C after collection and once thawed, centrifuged for 5 min at a relative centrifugal field of 300× *g*) of HEK-pNF-κB-d2EGFP/Neo L2 and HEK shRNA RelA cells, the LUNARIS^TM^ Human 11-Plex Cytokine Kit was used (LHCY-20110S, Ayoxxa Biosystems GmbH, Köln, Germany) which is based on beads-on-a-chip technology with a classical sandwich immunoassay principle and a fluorescence readout. The calibration curve was generated according to the kit handbook. Briefly, seven standards were prepared by serial dilution (1:4) of the Human Cytokine Standard using Assay Diluent 2. The standard contained the following analytes: GM-CSF, IFN-γ, IL-1β, IL-2, IL-4, IL-5, IL-6, IL-8, IL-10, IL-12(p70) and TNF-α. The LUNARIS^TM^ BioChips were first washed and the 5 µL of standard, blank and samples which were diluted 1:2 in Assay Diluent 2 were loaded into the appropriate wells. After an incubation of at least 3 h at room temperature, the BioChips were washed and 10 µL of detection antibody solition was added and allowed to incubate for 60 min at RT. The BioChips were washed again and then 10 µL of Streptavidin-Phycoerythrin (SA-PE) reagent was added. After 30 min of incubation, the final washing step was performed and the BioChips were air-dried and imaged using the LUNARIS^TM^ Reader (LRS-001). The quantification of the readout was performed using the LUNARIS^TM^ Analysis Suite Software.

### 4.11. Statistics

Each experiment was repeated up to five times depending on the availability of beam time with one to six replicates each. In order to account for different numbers of replicates and repeats, the standard error was calculated. Means, standard errors and significance levels in the t-test were calculated with Microsoft^®^ Office Excel 2010 (Microsoft Deutschland GmbH, München, Germany). Regression analyses were performed using SigmaPlot 13.0.

## Figures and Tables

**Table 1:** Basal gene expression in HEK shRNA RelA cells compared to the original cell line HEK-pNF-κB-d2EGFP/Neo L2 using the RT2 Profiler PCR array *.

Gene Symbol	Description	Relative Gene Expression (µ ± SE)
*CCL5*	Chemokine (C-C motif) ligand 5	15.57 ± 8.77
*CCND1*	Cyclin D1	−2.99 ± 0.05
*IL8/CXCL8*	Interleukin-8	−8.12 ± 5.20
*GADD45B*	Growth arrest and DNA-damage-inducible, beta	3.63 ± 1.08
*JUN*	Jun oncogene	4.96 ± 1.85
*RELA*	V-rel reticuloendotheliosis viral oncogene homolog A	−4.93 ± 0.02 **

* The table shows all genes from both the customized and the NF-κB signaling array, that are more than threefold up- or downregulated in HEK shRNA RelA cells compared to HEK-pNF-κB- d2EGFP/Neo L2 cells. 162 genes were investigated. Mean (µ) and standard error (SE) of the expression level were calculated from up to five independent experiments with untreated cells. Prior to investigating gene expression with RT^2^ Profiler^TM^ PCR arrays, a proper RNA and cDNA quality was confirmed. ** Five times less RelA mRNA in the HEK shRNA RelA cells compared to the HEK-pNF-κB-d2EGFP/Neo L2 cells results in a reduction of the expression level of 100% to 20% with a downregulation of 80%. This knock-down level is in line with the 87% observed using the RelA primer assays before. The complete data set is shown in Table A3.

**Table 2:** TNF-α * induced gene expression in HEK shRNA RelA cells (RelA k.d.) compared to the original cell line HEK-pNF-κB-d2EGFP/Neo L2 (HEK wt) using the RT2 Profiler PCR array **.

Gene Symbol	Description	Relative Gene Expression	
		HEK wt	RelA k.d.
*BIRC3*	Baculoviral IAP repeat containing 3	4.76 ± 0.98	2.03 ± 0.47
*C3*	Complement component 3	5.97 ± 1.39	1.97 ± 0.57
*CCL2*	Chemokine (C-C motif) ligand 2	3.04 ± 0.24	−1.28 ± 0.09
*CCL11*	Chemokine (C-C motif) ligand 11	3.73 ± 1.51	1.84 ± 0.57
*CCL5*	Chemokine (C-C motif) ligand 5	4.79 ± 1.79	29.02 ±12.66
*CD69*	CD69 molecule	3.37 ± 0.23	−1.15 ± 0.04
*CD83*	CD83 molecule	5.68 ± 0.82	1.67 ± 0.08
*CXCL1*	Chemokine (C-X-C motif) ligand 1	47.46 ± 11.75	−1.05 ± 0.08
*CXCL10*	Chemokine (C-X-C motif) ligand 10	23.38 ± 5.72	−1.18 ± 0.06
*CXCL2*	Chemokine (C-X-C motif) ligand 2	18.29 ± 3.92	1.72 ± 0.33
*EXO1*	Exonuclease 1	1.97 ± 0.67	−4.92 ± 0.02
*IL1R2*	Interleukin 1 receptor, type II	4.02 ± 1.29	1.71 ± 0.30
*IL8/CXCL8*	Interleukin-8	17.83 ± 5.19	2.39 ± 0.50
*IRF1*	Interferon regulatory factor 1	3.16 ± 0.40	1.29 ± 0.11
*MMP9*	Matrix metallopeptidase 9	3.34 ± 0.61	2.01 ± 0.37
*NFKBIA*	Nuclear factor of kappa light polypeptide gene enhancer in B-cells inhibitor, alpha	11.88 ± 1.66	1.22 ± 0.04
*PLAU*	Plasminogen activator, urokinase	3.35 ± 0.57	2.64 ± 0.58
*PTGS2*	Prostaglandin-endoperoxide synthase 2	3.99 ± 0.53	2.17 ± 0.39
*TNF*	Tumor necrosis factor	60.95 ± 12.42	3.40 ± 0.54
*TNFRSF1B*	Tumor necrosis factor receptor superfamily, member 1B	1.49 ± 0.34	4.13 ± 2.29

* Cells were treated for 6 h with 10 ng/mL TNF-α. ** The table shows all genes from both, the customized and the NF-κB signaling array, that are more than threefold up- or downregulated (in bold) in HEK shRNA RelA cells compared to HEK-pNF-kB-d2EGFP/Neo L2 cells. 162 genes were investigated. The mean of the fold up- or downregulation was calculated from up to two independent experiments with untreated cells. Prior to investigating gene expression with RT^2^ Profiler^TM^ PCR arrays, a proper RNA and cDNA quality was confirmed. The complete data set is shown in Table A4.

**Table 3:** Parameters of the survival curves *.

		D_0_ (Gy)		*n*		D_q_ (Gy)		Significance Level for D_0_
Radiation Quality	LET (keV/µm)	HEK wt	RelA k.d.	HEK wt	RelA k.d.	HEK wt	RelA k.d.	*p* = 2α
X-rays	0.3–3	1.20 ± 0.05	0.76 ± 0.04	1.31 ± 0.84	2.43 ± 1.06	0.33 ± 0.71	0.68 ± 0.33	<0.005
^12^C	10	1.03 ± 0.14	0.91 ± 0.22	1.17 ± 0.34	1.64 ± 1.15	0.15 ± 0.36	0.34 ± 0.71	0.6006
^28^Si	55	0.47 ± 0.05	0.38 ± 0.04	1.28 ± 0.34	1.31 ± 0.12	0.12 ± 0.14	0.10 ± 0.12	0.1923
^58^Ni	175	0.53 ± 0.02	0.61 ± 0.04	1.0 ± 0.26	0.73 ± 0.26	0.0 ± 0.12	−0.19 ± 0.19	0.0607
^36^Ar	271	0.72 ± 0.09	0.68 ± 0.08	0.82 ± 0.36	1.11 ± 0.29	−0.14 ± 0.27	0.08 ± 0.19	0.8371

* For cellular survival data the parameters D_0_ and n of the dose effect curve were calculated by regression analysis (survival versus dose) and fitted to the equation S = 1 − (1 – exp^D/Do^)*^n^*. The comparison of different survival curves were performed by Student’s t-test of the regression coefficients. A *p* = 2α level of <0.05 was considered as significant. Comparison of two regression lines for HEK-pNF-κB-d2EGFP/Neo L2 and HEK shRNA RelA cells is based on the hypothesis (σ^2^yx)_1_ ≠ (σ^2^yx)_2_ for D_0_; P, probability.

**Table 4:** Relative gene expression in HEK-pNF-κB-d2EGFP/Neo L2 (HEK wt) and HEK shRNA RelA (RelA k.d.) cells after irradiation * using the RT2 Profiler PCR array **. The complete data sets are shown in Table A5 and Table A6.

Radiation Quality	X-rays						^48^Ti ions			
Dose (Gy)	0.5		4.0		8.0		0.5		4.0	
Gene Symbol/Cell Line	HEK wt	RelA k.d.	HEK wt	RelA k.d.	HEK wt	RelA k.d.	HEK wt	RelA k.d.	HEK wt	RelA k.d.
*CCL5*	−1.42 ± 0.24	1.55 ± 0.68	3.68 ± 1.53	2.07 ± 0.90	3.34 ± 1.40	−1.47 ± 0.30	−1.97 ± 0.17	−1.89 ± 0.23	−1.56 ± 0.22	1.22 ± 0.53
*CD83*	−1.01 ± 0.03	1.12 ± 0.06	2.47 ± 0.08	1.67 ± 0.08	3.75 ± 0.18	2.27 ± 0.11	1.25 ± 0.04	1.09 ± 0.05	4.37 ± 0.14	1.42 ± 0.07
*CXCL1*	1.04 ± 0.20	1.06 ± 0.09	3.57 ± 0.56	1.52 ± 0.12	6.96 ± 1.01	2.02 ± 0.16	1.01 ± 0.19	−1.48 ± 0.05	7.55 ± 1.44	1.21 ± 0.10
*CXCL2*	−1.35 ± 0.13	−1.04 ± 0.19	2.33 ± 0.33	1.55 ± 0.30	3.01 ± 0.40	1.29 ± 0.25	1.30 ± 0.22	1.11 ± 0.21	3.48 ± 0.59	1.00 ± 0.19
*CXCL10*	−1.10 ± 0.16	1.05 ± 0.08	4.68 ± 0.60	2.23 ± 0.16	14.58± 2.57	2.04 ± 0.15	−1.27 ± 0.14	−1.30 ± 0.06	6.70 ± 1.20	−1.15 ± 0.06
*CXCL8/IL8*	−1.73 ± 0.19	1.20 ± 0.19	2.42 ± 0.66	1.38 ± 0.22	3.74 ± 0.86	1.01 ± 0.16	1.38 ± 0.44	−1.26 ± 0.13	6.10 ± 1.96	1.44 ± 0.23
*NFKB2*	1.01 ±0.12	1.09 ± 0.18	2.93 ± 0.33	1.48 ± 0.24	4.07 ± 0.45	1.62 ± 0.26	−1.25 ± 0.09	−1.02 ± 0.16	2.52 ± 0.29	1.04 ± 0.17
*NFKBIA*	−1.07 ± 0.08	−1.13 ± 0.10	2.65 ± 0.34	1.47 ± 0.17	4.26 ± 0.40	1.69 ± 0.19	−1.11 ± 0.08	1.05 ± 0.12	3.55 ± 0.31	1.47 ± 0.17
*TNF*	1.01 ± 0.11	1.08 ± 0.05	5.10 ± 0.99	1.17 ± 0.06	12.90 ± 1.72	1.15 ± 0.05	1.08 ± 0.12	−1.26 ± 0.04	6.72 ± 0.76	−1.12 ± 0.04
*TNFRSF1B*	−1.81 ± 0.16	−2.15 ± 0.26	1.02 ± 0.22	4.10 ± 2.28	1.11 ± 0.22	2.39 ± 1.33	1.07 ± 0.30	2.88 ± 1.60	−2.04 ± 0.14	−2.65 ± 0.21
*VCAM1*	−1.27 ± 0.20	1.08 ± 0.05	4.24 ± 1.56	1.17 ± 0.06	2.94 ± 0.87	1.15 ± 0.05	−1.77 ± 0.15	−1.26 ± 0.04	−1.10 ± 0.24	−1.12 ± 0.04

* Exposure to X-rays or ^48^Ti-ions (1000 MeV/n, LET 108 keV/µm). ** The table shows all genes from the NF-κB signaling array, that are more than threefold up- or down-regulated (in bold) in irradiated cells compared to mock-irradiated cells. The mean of the fold up- or downregulation was calculated from up to two independent experiments with untreated cells. Prior to investigating gene expression with RT2 Profiler^TM^ PCR arrays, a proper RNA and cDNA quality was confirmed. ACTB, B2M, GAPDH, HPRT1 and RPLP0 were used as reference genes to normalize the results.

**Table 5:** Characteristics of the heavy ion beams.

	Energy (MeV/n) ^a^		LET (keV/µm)	Penetration Depth (µm)	Accelerator
Ion Species	Beam	on Target ^b^	in H_2_O		
Carbon (^12^C)	400.0	400.0	10.0	272,900	HIMAC
Carbon (^13^C)	75.0	71.4	34.2	15,120	GANIL
Silicon (^28^Si)	490.0	490.0	55.0	161,300	HIMAC
Titanium (^48^Ti)	1000.0	996.9	107.7	319,900	GSI
Nickel (^58^Ni)	1000.0	996.9	174.5	263,600	GSI
Argon (^36^Ar)	95.0	83.8	271.5	6336	GANIL

^a^ Mega-electron-Volt/nucleon. ^b^ Effective irradiation energy at the cell monolayer. For GANIL beam times energy losses occur in two detectors, the exit window, ~1 cm air and the bottom of the culture vessel (1200 µm polystyrene or 25 µm polytetrafluoroethylene foil). For GSI and HIMAC beams energy losses are marginal (only from bottom of the culture vessel).

**Table 6:** RT-qPCR primer pairs * as described in the RT2 qPCR Primer Assay (SABiosciences).

Gene Symbol	REF SEQ ACC. #	Amplicon Size	Melting Temperature
RELA	NM_021975.3	65 bp	80.0 °C
HPRT1	NM_000194.2	89 bp	76.5 °C

* All genes listed are human genes. HPRT, Hypoxanthine phosphoribosyltransferase 1; RELA, V-rel reticuloendotheliosis viral oncogene homolog A (avian).

## Abbreviations

AP-1	activated protein 1
ATCC	American Type Culture Collection
CIMAP	Centre de recherches sur les Ions, les Matériaux et la Photonique
d2EGFP	destabilized EGFP
DDR	DNA damage response
DNA-PK	DNA-dependent protein kinase
DSB	double strand break
EGFP	Enhanced Green Fluorescent Protein
ESCC	esophageal squamous cell cancer
EXO1	exonuclease 1
FBS	fetal bovine serum
GANIL	“Grand Accélérateur National d’Ions Lourds”
GSI	GSI Helmholtzzentrum für Schwerionenforschung GmbH
HEK	human embryonic kidney
HIMAC	Heavy Ion Medical Accelerator in Chiba
HR	Homologous Recombination
HSPC	hematopoietic stem and progenitor cells
IAP	inhibitor of apoptosis protein
IκBα	inhibitor of NF-κB α
IKK	IκB kinase
IκBα-SR	super-repressor variant of IκBα
ISS	International Space Station
k.d.	knock-down
LARIA	“Laboratoire d’Accueil en Radiobiologie avec les Ions Accélérés”
LET	linear energy transfer
MDR1	multi-drug resistance
MeV/n	Mega electron Volt per nucleon
MnSOD	manganese superoxide dismutase
mRNA	messenger RNA
NF-κB	Nuclear factor κB
NHEJ	Non-Homologous Endjoining
NPCs	neural progenitor cells
NRE	NF-κB response element
NSCs	neural stem cells
P	probability
PBL	peripheral blood lymphocytes
PBS	phosphate buffered saline
PI3K	phosphatidyl inositol kinase 3
RBE	relative biologic effectiveness
Rel	Reticuloendotheliosis
RT-qPCR	Reverse Transcriptase real-time quantitative PCR
shRNA RelA	RelA short-hairpin RNA
SD	standard deviation
SE	standard error
TK	thymidine kinase
TLR5	Toll-like receptor 5
TNF-α	tumor necrosis factor α
TP53	tumor protein p53

## Appendix A

**Table A1:** Gene list of QPCR Array PAHS-225ZD.

UniGene	GeneBank	Symbol	Description	Gene Names
Hs.441047	NM_001124	*ADM*	Adrenomedullin	*AM*
Hs.19383	NM_000029	*AGT*	Angiotensinogen (serpin peptidase inhibitor, clade A, member 8)	*ANHU*, *FLJ92595*, *FLJ97926*, *SERPINA8*
Hs.525622	NM_005163	*AKT1*	V-akt murine thymoma viral oncogene homolog 1	*AKT*, *MGC99656*, *PKB*, *PKB-ALPHA*, *PRKBA*, *RAC*, *RAC-ALPHA*
Hs.499886	NM_000382	*ALDH3A2*	Aldehyde dehydrogenase 3 family, member A2	*ALDH10*, *DKFZp686E23276*, *FALDH*, *FLJ20851*, *SLS*
Hs.227817	NM_004049	*BCL2A1*	BCL2-related protein A1	*ACC-1*, *ACC-2*, *BCL2L5*, *BFL1*, *GRS*, *HBPA1*
Hs.516966	NM_138578	*BCL2L1*	BCL2-like 1	*BCL-XL*, *S*, *BCL2L*, *BCLX*, *BCLXL*, *BCLXS*, *Bcl-X*, *DKFZp781P2092*, *bcl-xL*, *bcl-xS*
Hs.696238	NM_001166	*BIRC2*	Baculoviral IAP repeat containing 2	*API1*, *HIAP2*, *Hiap-2*, *MIHB*, *RNF48*, *c-IAP1*, *cIAP1*
Hs.127799	NM_001165	*BIRC3*	Baculoviral IAP repeat containing 3	*AIP1*, *API2*, *CIAP2*, *HAIP1*, *HIAP1*, *MALT2*, *MIHC*, *RNF49*, *c-IAP2*
Hs.529053	NM_000064	*C3*	Complement component 3	*AHUS5*, *ARMD9*, *ASP*, *CPAMD1*
Hs.54460	NM_002986	*CCL11*	Chemokine (C-C motif) ligand 11	*MGC22554*, *SCYA11*
Hs.303649	NM_002982	*CCL2*	Chemokine (C-C motif) ligand 2	*GDCF-2*, *HC11*, *HSMCR30*, *MCAF*, *MCP-1*, *MCP1*, *MGC9434*, *SCYA2*, *SMC-CF*
Hs.534347	NM_002990	*CCL22*	Chemokine (C-C motif) ligand 22	*ABCD-1*, *DC*, *B-CK*, *MDC*, *MGC34554*, *SCYA22*, *STCP-1*
Hs.514821	NM_002985	*CCL5*	Chemokine (C-C motif) ligand 5	*D17S136E*, *MGC17164*, *RANTES*, *SCYA5*, *SISd*, *TCP228*
Hs.523852	NM_053056	*CCND1*	Cyclin D1	*BCL1*, *D11S287E*, *PRAD1*, *U21B31*
Hs.450802	NM_000579	*CCR5*	Chemokine (C-C motif) receptor 5	*CC-CKR-5*, *CCCKR5*, *CD195*, *CKR-5*, *CKR5*, *CMKBR5*, *FLJ78003*, *IDDM22*
Hs.472860	NM_001250	*CD40*	CD40 molecule, TNF receptor superfamily member 5	*Bp50*, *CDW40*, *MGC9013*, *TNFRSF5*, *p50*
Hs.208854	NM_001781	*CD69*	CD69 molecule	*CLEC2C*
Hs.838	NM_005191	*CD80*	CD80 molecule	*B7*, *B7-1*, *B7.1*, *BB1*, *CD28LG*, *CD28LG1*, *LAB7*
Hs.595133	NM_004233	*CD83*	CD83 molecule	*BL11*, *HB15*
Hs.370771	NM_000389	*CDKN1A*	Cyclin-dependent kinase inhibitor 1A (p21, Cip1)	*CAP20*, *CDKN1*, *CIP1*, *MDA-6*, *P21*, *SDI1*, *WAF1*, *p21CIP1*
Hs.69771	NM_001710	*CFB*	Complement factor B	*AHUS4*, *BF*, *BFD*, *CFAB*, *FB*, *FBI12*, *FLJ54899*, *GBG*, *H2-Bf*, *PBF2*
Hs.591402	NM_000757	*CSF1*	Colony stimulating factor 1 (macrophage)	*MCSF*, *MGC31930*
Hs.1349	NM_000758	*CSF2*	Colony stimulating factor 2 (granulocyte-macrophage)	*GMCSF*, *MGC131935*, *MGC138897*
Hs.592192	NM_000395	*CSF2RB*	Colony stimulating factor 2 receptor, beta, low-affinity (granulocyte-macrophage)	*CD131*, *CDw131*, *IL3RB*, *IL5RB*
Hs.2233	NM_000759	*CSF3*	Colony stimulating factor 3 (granulocyte)	*C17orf33*, *CSF3OS*, *GCSF*, *MGC45931*
Hs.789	NM_001511	*CXCL1*	Chemokine (C-X-C motif) ligand 1 (melanoma growth stimulating activity, alpha)	*FSP*, *GRO1*, *GROa*, *MGSA*, *MGSA-a*, *NAP-3*, *SCYB1*
Hs.632586	NM_001565	*CXCL10*	Chemokine (C-X-C motif) ligand 10	*C7*, *IFI10*, *INP10*, *IP-10*, *SCYB10*, *crg-2*, *gIP-10*, *mob-1*
Hs.590921	NM_002089	*CXCL2*	Chemokine (C-X-C motif) ligand 2	*CINC-2a*, *GRO2*, *GROb*, *MGSA-b*, *MIP-2a*, *MIP2*, *MIP2A*, *SCYB2*
Hs.77367	NM_002416	*CXCL9*	Chemokine (C-X-C motif) ligand 9	*CMK*, *Humig*, *MIG*, *SCYB9*, *crg-10*
Hs.488293	NM_005228	*EGFR*	Epidermal growth factor receptor	*ERBB*, *ERBB1*, *HER1*, *PIG61*, *mENA*
Hs.1395	NM_000399	*EGR2*	Early growth response 2	*AT591*, *CMT1D*, *CMT4E*, *DKFZp686J1957*, *FLJ14547*, *KROX20*
Hs.62192	NM_001993	*F3*	Coagulation factor III (thromboplastin, tissue factor)	*CD142*, *FLJ17960*, *TF*, *TFA*
Hs.654450	NM_000132	*F8*	Coagulation factor VIII, procoagulant component	*AHF*, *DXS1253E*, *F8B*, *F8C*, *FVIII*, *HEMA*
Hs.244139	NM_000043	*FAS*	Fas (TNF receptor superfamily, member 6)	*ALPS1A*, *APO-1*, *APT1*, *CD95*, *FAS1*, *FASTM*, *TNFRSF6*
Hs.2007	NM_000639	*FASLG*	Fas ligand (TNF superfamily, member 6)	*APT1LG1*, *CD178*, *CD95-L*, *CD95L*, *FASL*, *TNFSF6*
Hs.110571	NM_015675	*GADD45B*	Growth arrest and DNA-damage-inducible, beta	*DKFZp566B133*, *GADD45BETA*, *MYD118*
Hs.643447	NM_000201	*ICAM1*	Intercellular adhesion molecule 1	*BB2*, *CD54*, *P3.58*
Hs.93177	NM_002176	*IFNB1*	Interferon, beta 1, fibroblast	*IFB*, *IFF*, *IFNB*, *MGC96956*
Hs.856	NM_000619	*IFNG*	Interferon, gamma	*IFG*, *IFI*
Hs.674	NM_002187	*IL12B*	Interleukin 12B (natural killer cell stimulatory factor 2, cytotoxic lymphocyte maturation factor 2, p40)	*CLMF*, *CLMF2*, *IL-12B*, *NKSF*, *NKSF2*
Hs.654378	NM_000585	*IL15*	Interleukin 15	*IL-15*, *MGC9721*
Hs.1722	NM_000575	*IL1A*	Interleukin 1, alpha	*IL-1A*, *IL1*, *IL1-ALPHA*, *IL1F1*
Hs.126256	NM_000576	*IL1B*	Interleukin 1, beta	*IL-1*, *IL1-BETA*, *IL1F2*
Hs.25333	NM_004633	*IL1R2*	Interleukin 1 receptor, type II	*CD121b*, *IL1RB*, *MGC47725*
Hs.81134	NM_000577	*IL1RN*	Interleukin 1 receptor antagonist	*DIRA*, *ICIL-1RA*, *IL-1RN*, *IL-1ra*, *IL-1ra3*, *IL1F3*, *IL1RA*, *IRAP*, *MGC10430*, *MVCD4*
Hs.89679	NM_000586	*IL2*	Interleukin 2	*IL-2*, *TCGF*, *lymphokine*
Hs.231367	NM_000417	*IL2RA*	Interleukin 2 receptor, alpha	*CD25*, *IDDM10*, *IL2R*, *TCGFR*
Hs.73917	NM_000589	*IL4*	Interleukin 4	*BCGF-1*, *BCGF1*, *BSF-1*, *BSF1*, *IL-4*, *MGC79402*
Hs.654458	NM_000600	*IL6*	Interleukin 6 (interferon, beta 2)	*BSF2*, *HGF*, *HSF*, *IFNB2*, *IL-6*
Hs.624	NM_000584	*IL8*	Interleukin 8	*CXCL8*, *GCP-1*, *GCP1*, *LECT*, *LUCT*, *LYNAP*, *MDNCF*, *MONAP*, *NAF*, *NAP-1*, *NAP1*
Hs.654579	NM_000207	*INS*	Insulin	*IDDM2*, *ILPR*, *IRDN*, *MODY10*
Hs.436061	NM_002198	*IRF1*	Interferon regulatory factor 1	*IRF-1*, *MAR*
Hs.36	NM_000595	*LTA*	Lymphotoxin alpha (TNF superfamily, member 1)	*LT*, *TNFB*, *TNFSF1*
Hs.376208	NM_002341	*LTB*	Lymphotoxin beta (TNF superfamily, member 3)	*TNFC*, *TNFSF3*, *p33*
Hs.463978	NM_002758	*MAP2K6*	Mitogen-activated protein kinase kinase 6	*MAPKK6*, *MEK6*, *MKK6*, *PRKMK6*, *SAPKK3*
Hs.297413	NM_004994	*MMP9*	Matrix metallopeptidase 9 (gelatinase B, 92kDa gelatinase, 92kDa type IV collagenase)	*CLG4B*, *GELB*, *MANDP2*, *MMP-9*
Hs.202453	NM_002467	*MYC*	V-myc myelocytomatosis viral oncogene homolog (avian)	*MRTL*, *bHLHe39*, *c-Myc*
Hs.82116	NM_002468	*MYD88*	Myeloid differentiation primary response gene (88)	*MYD88D*
Hs.592142	NM_181659	*NCOA3*	Nuclear receptor coactivator 3	*ACTR*, *AIB-1*, *AIB1*, *CAGH16*, *CTG26*, *KAT13B*, *MGC141848*, *RAC3*, *SRC-3*, *SRC3*, *TNRC14*, *TNRC16*, *TRAM-1*, *bHLHe42*, *pCIP*
Hs.654408	NM_003998	*NFKB1*	Nuclear factor of kappa light polypeptide gene enhancer in B-cells 1	*DKFZp686C01211*, *EBP-1*, *KBF1*, *MGC54151*, *NF-kappa-B*, *NF-kappaB*, *NFKB-p105*, *NFKB-p50*, *NFkappaB*, *p105*, *p50*
Hs.73090	NM_002502	*NFKB2*	Nuclear factor of kappa light polypeptide gene enhancer in B-cells 2 (p49/p100)	*LYT-10*, *LYT10*, *p52*
Hs.81328	NM_020529	*NFKBIA*	Nuclear factor of kappa light polypeptide gene enhancer in B-cells inhibitor, alpha	*IKBA*, *MAD-3*, *NFKBI*
Hs.406515	NM_000903	*NQO1*	NAD(P)H dehydrogenase, quinone 1	*DHQU*, *DIA4*, *DTD*, *NMOR1*, *NMORI*, *QR1*
Hs.563344	NM_006186	*NR4A2*	Nuclear receptor subfamily 4, group A, member 2	*HZF-3*, *NOT*, *NURR1*, *RNR1*, *TINUR*
Hs.1976	NM_002608	*PDGFB*	Platelet-derived growth factor beta polypeptide	*FLJ12858*, *PDGF2*, *SIS*, *SSV*, *c-sis*
Hs.77274	NM_002658	*PLAU*	Plasminogen activator, urokinase	*ATF*, *UPA*, *URK*, *u-PA*
Hs.196384	NM_000963	*PTGS2*	Prostaglandin-endoperoxide synthase 2 (prostaglandin G/H synthase and cyclooxygenase)	*COX-2*, *COX2*, *GRIPGHS*, *PGG*, *HS*, *PGHS-2*, *PHS-2*, *hCox-2*
Hs.631886	NM_002908	*REL*	V-rel reticuloendotheliosis viral oncogene homolog (avian)	*C-Rel*
Hs.502875	NM_021975	*RELA*	V-rel reticuloendotheliosis viral oncogene homolog A (avian)	*MGC131774*, *NFKB3*, *p65*
Hs.654402	NM_006509	*RELB*	V-rel reticuloendotheliosis viral oncogene homolog B	*I-REL*, *IREL*, *REL-B*
Hs.89546	NM_000450	*SELE*	Selectin E	*CD62E*, *ELAM*, *ELAM1*, *ESEL*, *LECAM2*
Hs.73800	NM_003005	*SELP*	Selectin P (granule membrane protein 140kDa, antigen CD62)	*CD62*, *CD62P*, *FLJ45155*, *GMP140*, *GRMP*, *LECAM3*, *PADGEM*, *PSEL*
Hs.167317	NM_003081	*SNAP25*	Synaptosomal-associated protein, 25kDa	*FLJ23079*, *RIC-4*, *RIC4*, *SEC9*, *SNAP*, *SNAP-25*, *bA416N4.2*, *dJ1068F16.2*
Hs.487046	NM_000636	*SOD2*	Superoxide dismutase 2, mitochondrial	*IPOB*, *MNSOD*, *MVCD6*
Hs.642990	NM_007315	*STAT1*	Signal transducer and activator of transcription 1, 91kDa	*DKFZp686B04100*, *ISGF-3*, *STAT91*
Hs.463059	NM_003150	*STAT3*	Signal transducer and activator of transcription 3 (acute-phase response factor)	*APRF*, *FLJ20882*, *HIES*, *MGC16063*
Hs.595276	NM_012448	*STAT5B*	Signal transducer and activator of transcription 5B	*STAT5*
Hs.241570	NM_000594	*TNF*	Tumor necrosis factor	*DIF*, *TNF-alpha*, *TNFA*, *TNFSF2*
Hs.256278	NM_001066	*TNFRSF1B*	Tumor necrosis factor receptor superfamily, member 1B	*CD120b*, *TBPII*, *TNF-R-II*, *TNF-R75*, *TNFBR*, *TNFR1B*, *TNFR2*, *TNFR80*, *p75*, *p75TNFR*
Hs.478275	NM_003810	*TNFSF10*	Tumor necrosis factor (ligand) superfamily, member 10	*APO2L*, *Apo-2L*, *CD253*, *TL2*, *TRAIL*
Hs.654481	NM_000546	*TP53*	Tumor protein p53	*FLJ92943*, *LFS1*, *P53*, *TRP53*
Hs.522506	NM_021138	*TRAF2*	TNF receptor-associated factor 2	*MGC:45012*, *TRAP*, *TRAP3*
Hs.109225	NM_001078	*VCAM1*	Vascular cell adhesion molecule 1	*CD106*, *DKFZp779G2333*, *INCAM-100*, *MGC99561*
Hs.356076	NM_001167	*XIAP*	X-linked inhibitor of apoptosis	*API3*, *BIRC4*, *FLJ26913*, *IAP-3*, *ILP1*, *MIHA*, *XLP2*, *hIAP-3*, *hIAP3*
Hs.520640	NM_001101	*ACTB*	Actin, beta	*PS1TP5BP1*
Hs.534255	NM_004048	*B2M*	Beta-2-microglobulin	*-*
Hs.592355	NM_002046	*GAPDH*	Glyceraldehyde-3-phosphate dehydrogenase	*G3PD*, *GAPD*, *MGC88685*
Hs.412707	NM_000194	*HPRT1*	Hypoxanthine phosphoribosyltransferase 1	*HGPRT*, *HPRT*
Hs.546285	NM_001002	*RPLP0*	Ribosomal protein, large, P0	*L10E*, *LP0*, *MGC111226*, *MGC88175*, *P0*, *PRLP0*, *RPP0*
N/A	SA_00105	*HGDC*	Human Genomic DNA Contamination	*HIGX1A*

**Table A2:** List Additional Genes of the Customized QPCR Array.

UniGene	GeneBank	Symbol	Description	Gene Names
Hs.131431	NM_002759	*EIF2AK2*	Eukaryotic translation initiation factor 2-alpha kinase 2	*EIF2AK1/PKR*
Hs.141125	NM_004346	*CASP3*	Caspase 3, apoptosis-related cysteine peptidase	*CPP32/CPP32B*
Hs.146806	NM_003592	*CUL1*	Cullin 1	*MGC149834*
Hs.147433	NM_182649	*PCNA*	Proliferating cell nuclear antigen	*MGC8367*
Hs.160953	NM_022112	*P53AIP1*	P53-regulated apoptosis-inducing protein 1	*P53AIP1*
Hs.1770	NM_000234	*LIG1*	Ligase I, DNA, ATP-dependent	*MGC117397*
Hs.191334	NM_003362	*UNG*	Uracil-DNA glycosylase	*DGU/DKFZp781L1143*
Hs.191356	NM_001515	*GTF2H2*	General transcription factor IIH, polypeptide 2, 44 kDa	*BTF2/BTF2P44*
Hs.193717	NM_000572	*IL10*	Interleukin 10	*CSIF/IL-10*
Hs.194143	NM_007294	*BRCA1*	Breast cancer 1, early onset	*BRCAI/BRCC1*
Hs.2420	NM_003102	*SOD3*	Superoxide dismutase 3, extracellular	*EC-SOD*
Hs.244723	NM_001238	*CCNE1*	Cyclin E1	*CCNE*
Hs.24529	NM_001274	*CHEK1*	CHK1 checkpoint homolog (*S. pombe*)	*CHK1*
Hs.271791	NM_001184	*ATR*	Ataxia telangiectasia and Rad3 related	*FRP1/MEC1*
Hs.290758	NM_001923	*DDB1*	Damage-specific DNA binding protein 1, 127 kDa	*DDBA/UV-DDB1*
Hs.291363	NM_007194	*CHEK2*	CHK2 checkpoint homolog (*S. pombe*)	*CDS1/CHK2*
Hs.31210	NM_005178	*BCL3*	B-cell CLL/lymphoma 3	*BCL4/D19S37*
Hs.321045	NM_014002	*IKBKE*	Inhibitor of kappa light polypeptide gene enhancer in B-cells, kinase epsilon	*IKK-i/IKKE*
Hs.326035	NM_001964	*EGR1*	Early growth response 1	*AT225/G0S30*
Hs.329502	NM_001229	*CASP9*	Caspase 9, apoptosis-related cysteine peptidase	*APAF-3/APAF3*
Hs.334562	NM_001786	*CDC2*	Cell division cycle 2, G1 to S and G2 to M	*CDC28A/CDK1*
Hs.34012	NM_000059	*BRCA2*	Breast cancer 2, early onset	*BRCC2/FACD*
Hs.367437	NM_000051	*ATM*	Ataxia telangiectasia mutated	*AT1/ATA*
Hs.380271	NM_002542	*OGG1*	8-oxoguanine DNA glycosylase	*HMMH/HOGG1*
Hs.388739	NM_021141	*XRCC5*	X-ray repair complementing defective repair in Chinese hamster cells 5 (double-strand-break rejoining; Ku autoantigen, 80 kDa)	*KARP-1/KARP1*
Hs.408528	NM_000321	*RB1*	Retinoblastoma 1 (including osteosarcoma)	*OSRC/RB*
Hs.431850	NM_002745	*MAPK1*	Mitogen-activated protein kinase 1	*ERK/ERK2*
Hs.43505	NM_003639	*IKBKG*	Inhibitor of kappa light polypeptide gene enhancer in B-cells, kinase gamma	*AMCBX1/FIP-3*
Hs.437705	NM_001789	*CDC25A*	Cell division cycle 25 homolog A (*S. pombe*)	*CDC25A2*
Hs.443914	NM_000454	*SOD1*	Superoxide dismutase 1, soluble (amyotrophic lateral sclerosis 1 (adult))	*ALS/ALS1*
Hs.445203	NM_001539	*DNAJA1*	DnaJ (Hsp40) homolog, subfamily A, member 1	*DJ-2/DjA1*
Hs.460996	NM_003789	*TRADD*	TNFRSF1A-associated via death domain	*Hs.89862*
Hs.469872	NM_000122	*ERCC3*	Excision repair cross-complementing rodent repair deficiency, complementation group 3 (xeroderma pigmentosum group B complementing)	*BTF2/GTF2H*
Hs.492208	NM_002485	*NBN*	Nibrin	*AT-V1/AT-V2*
Hs.498248	NM_130398	*EXO1*	Exonuclease 1	*HEX1/hExoI*
Hs.502302	NM_001752	*CAT*	Catalase	*MGC138422*
Hs.505033	NM_004985	*KRAS*	V-Ki-ras2 Kirsten rat sarcoma viral oncogene homolog	*C-K-RAS/K-RAS2A*
Hs.505777	NM_004083	*DDIT3*	DNA-damage-inducible transcript 3	*CEBPZ/CHOP*
Hs.512599	NM_000077	*CDKN2A*	Cyclin-dependent kinase inhibitor 2A (melanoma, p16, inhibits CDK4)	*ARF/CDK4I*
Hs.514527	NM_001168	*BIRC5*	Baculoviral IAP repeat-containing 5 (survivin)	*API4/EPR-1*
Hs.520028	NM_005345	*HSPA1A*	Heat shock 70 kDa protein 1A	*HSP70-1/HSP72*
Hs.523185	NM_012423	*RPL13A*	Ribosomal protein L13a	*RPL13A*
Hs.523560	NM_001040141	*HSP90AA2*	Heat shock protein 90 kDa alpha (cytosolic), class A member 2	*HSP90ALPHA/HSPCA*
Hs.523968	NM_005426	*TP53BP2*	Tumor protein p53 binding protein, 2	*53BP2/ASPP2*
Hs.525704	NM_002228	*JUN*	Jun oncogene	*AP1/c-Jun*
Hs.530227	NM_005526	*HSF1*	Heat shock transcription factor 1	*HSTF1*
Hs.531704	NM_002737	*PRKCA*	Protein kinase C, alpha	*AAG6/PKC-alpha*
Hs.544577	NM_002046	*GAPDH*	Glyceraldehyde-3-phosphate dehydrogenase	*G3PD/GAPD*
Hs.553498	NM_006218	*PIK3CA*	Phosphoinositide-3-kinase, catalytic, alpha polypeptide	*PI3K/p110-alpha*
Hs.567303	NM_002392	*MDM2*	Mdm2, transformed 3T3 cell double minute 2, p53 binding protein (mouse)	*HDMX/hdm2*
Hs.567387	NM_057749	*CCNE2*	Cyclin E2	*CYCE2*
Hs.591084	NM_004629	*FANCG*	Fanconi anemia, complementation group G	*FAG/XRCC9*
Hs.591980	NM_000378	*WT1*	Wilms tumor 1	*GUD/WAGR*
Hs.592325	NM_005432	*XRCC3*	X-ray repair complementing defective repair in Chinese hamster cells 3	*XRCC3*
Hs.592839	NM_005633	*SOS1*	Son of sevenless homolog 1 (Drosophila)	*GF1/GGF1*
Hs.61188	NM_033276	*XRCC6BP1*	XRCC6 binding protein 1	*KUB3*
Hs.631709	NM_002875	*RAD51*	RAD51 homolog (RecA homolog, E. coli) (S. cerevisiae)	*BRCC5/HRAD51*
Hs.647093	NM_005431	*XRCC2*	X-ray repair complementing defective repair in Chinese hamster cells 2	*DKFZp781P0919*
Hs.647371	NM_005953	*MT2A*	Metallothionein 2A	*MT2*
Hs.654393	NM_005225	*E2F1*	E2F transcription factor 1	*E2F-1/RBBP3*
Hs.654532	NM_003263	*TLR1*	Toll-like receptor 1	*CD281/DKFZp547I0610*
Hs.655354	NM_004584	*RAD9A*	RAD9 homolog A (*S. pombe*)	*RAD9*
Hs.655983	NM_001228	*CASP8*	Caspase 8, apoptosis-related cysteine peptidase	*ALPS2B/CAP4*
Hs.656458	NM_001556	*IKBKB*	Inhibitor of kappa light polypeptide gene enhancer in B-cells, kinase beta	*IKK-beta/IKK2*
Hs.695926	NM_002890	*RASA1*	RAS p21 protein activator (GTPase activating protein) 1	*CM-AVM/CMAVM*
Hs.697294	NM_005427	*TP73*	Tumor protein p73	*P73*
Hs.706746	NM_000625	*NOS2A*	Nitric oxide synthase 2A (inducible, hepatocytes)	*HEP-NOS/INOS*
Hs.708288	NM_000165	*GJA1*	Gap junction protein, alpha 1, 43 kDa	*CX43/DFNB38*
Hs.73133	NM_005954	*MT3*	Metallothionein 3	*GIF/GIFB*
Hs.76686	NM_000581	*GPX1*	Glutathione peroxidase 1	*GSHPX1*
Hs.80409	NM_001924	*GADD45A*	Growth arrest and DNA-damage-inducible, alpha	*DDIT1/GADD45*
Hs.87247	NM_003806	*HRK*	Harakiri, BCL2 interacting protein (contains only BH3 domain)	*DP5/HARAKIRI*
Hs.9701	NM_006705	*GADD45G*	Growth arrest and DNA-damage-inducible, gamma	*CR6/DDIT2*
Hs.98493	NM_006297	*XRCC1*	X-ray repair complementing defective repair in Chinese hamster cells 1	*RCC*

**Table A3:** Basal gene expression in HEK shRNA RelA cells compared to the original cell line HEK-pNF-κB-d2EGFP/Neo L2 using the RT2 Profiler PCR array.

Symbol	µ	SE	Symbol	µ	SE	Symbol	µ	SE
*ACTB*	1.02	0.06	*CFB*	−1.12	0.13	*HSP90AA2*	1.74	0.19
*ADM*	1.42	0.23	*CHEK1*	1.01	0.16	*HSPA1A*	1.86	0.33
*AGT*	−1.02	0.13	*CHEK2*	−1.11	0.06	*ICAM1*	1.13	0.16
*AKT1*	−1.11	0.10	*CSF1*	−1.24	0.06	*IFNB1*	−1.00	0.05
*ALDH3A2*	−1.15	0.03	*CSF2*	1.06	0.18	*IFNG*	−1.00	0.05
*ATM*	−1.79	0.02	*CSF2RB*	−1.00	0.05	*IKBKB*	−1.37	0.06
*ATR*	1.40	0.08	*CSF3*	1.35	0.20	*IKBKE*	−2.40	0.02
*B2M*	−1.03	0.07	*CUL1*	1.04	0.12	*IKBKG*	1.10	0.27
*BCL2A1*	1.06	0.11	*CXCL1*	−1.59	0.12	*IL10*	1.67	0.43
*BCL2L1*	1.08	0.13	*CXCL10*	−1.32	0.15	*IL12B*	−1.15	0.06
*BCL3*	−1.15	0.21	*CXCL2*	1.11	0.24	*IL15*	−1.36	0.11
*BIRC2*	−1.03	0.09	*CXCL9*	1.57	0.38	*IL1A*	1.12	0.17
*BIRC3*	1.23	0.30	*DDB1*	1.21	0.28	*IL1B*	1.09	0.08
*BIRC5*	1.06	0.10	*DDIT3*	1.62	0.51	*IL1R2*	−1.05	0.24
*BRCA2*	1.08	0.12	*DNAJA1*	−1.23	0.09	*IL1RN*	−1.00	0.05
*BRCA5*	−1.04	0.28	*E2F1*	1.27	0.40	*IL2*	−1.00	0.05
*C3*	1.88	0.69	*EGFR*	−1.04	0.11	*IL2RA*	−1.00	0.05
*CASP3*	1.27	0.09	*EGR1*	2.15	0.30	*IL4*	1.97	0.33
*CASP8*	−1.22	0.30	*EGR2*	1.30	0.30	*IL6*	1.27	0.28
*CASP9*	−1.07	0.06	*EIF2AK2*	−1.24	0.04	*IL8*	−2.31	0.13
*CAT*	1.03	0.22	*ERCC3*	−1.18	0.09	*INS*	−1.00	0.05
*CCL11*	1.22	0.32	*EXO1*	−1.19	0.21	*IRF1*	−1.11	0.08
*CCL2*	1.36	0.11	*F3*	1.20	0.15	*JUN*	4.96	1.85
*CCL22*	−1.00	0.05	*F8*	1.16	0.06	*KRAS*	−1.08	0.02
*CCL5*	15.57	8.77	*FANCG*	−1.48	0.04	*LIG1*	−1.18	0.23
*CCND1*	−2.99	0.05	*FAS*	−1.30	0.13	*LTA*	1.54	0.33
*CCNE1*	1.03	0.08	*FASLG*	2.50	0.54	*LTB*	1.09	0.25
*CCNE2*	1.18	0.23	*GADD45A*	−1.27	0.06	*MAP2K6*	−1.23	0.13
*CCR5*	1.53	0.28	*GADD45B*	3.63	1.08	*MAPK1*	−1.10	0.12
*CD40*	−1.02	0.15	*GADD45G*	−1.17	0.19	*MDM2*	−1.24	0.11
*CD69*	−1.00	0.05	*GAPDH*	1.13	0.07	*MMP9*	−1.03	0.23
*CD80*	1.18	0.26	*GJA1*	−1.22	0.16	*MT2A*	1.01	0.07
*CD83*	−1.50	0.09	*GPX1*	1.52	0.17	*MT3*	1.88	0.80
*CDC2*	1.12	0.21	*GTF2H2*	−1.24	0.06	*MYC*	1.14	0.20
*CDC25A*	1.22	0.10	*HPRT1*	1.21	0.08	*MYD88*	−1.33	0.17
*CDKN1A*	1.37	0.31	*HRK*	1.12	0.48	*NBN*	−2.37	0.09
*CDKN2A*	1.11	0.06	*HSF1*	1.06	0.21	*NCOA3*	1.33	0.06
*NFKB1*	1.00	0.06	*RB1*	1.00	0.04	*TNF*	−1.69	0.12
*NFKB2*	1.19	0.16	*REL*	−1.27	0.04	*TNFRSF1B*	1.02	0.25
*NFKBIA*	−1.64	0.08	*RELA*	−4.93	0.02	*TNFSF10*	−1.01	0.23
*NOS2*	1.40	0.32	*RELB*	1.14	0.22	*TP53*	−1.17	0.04
*NQO1*	1.28	0.19	*RPL13A*	−1.12	0.13	*TP53BP2*	−1.13	0.10
*NR4A2*	2.40	0.23	*RPLP0*	−1.19	0.06	*TP73*	1.02	0.09
*OGG1*	1.02	0.18	*SELE*	1.64	0.38	*TRADD*	−1.46	0.04
*P53AIP1*	1.45	0.30	*SELP*	−1.00	0.05	*TRAF2*	−1.12	0.03
*PCNA*	1.28	0.16	*SNAP25*	1.79	0.38	*UNG*	1.66	0.18
*PDGFB*	−1.00	0.05	*SOD1*	1.21	0.15	*VCAM1*	−1.00	0.05
*PIK3Ca*	1.13	0.07	*SOD2*	−1.09	0.06	*WT1*	1.25	0.19
*PLAU*	−1.52	0.14	*SOD3*	2.03	0.75	*XIAP*	−1.10	0.12
*PRKCA*	−1.03	0.12	*SOS1*	−1.11	0.13	*XRCC1*	1.01	0.09
*PTGS2*	1.96	0.38	*STAT1*	1.25	0.05	*XRCC2*	−1.52	0.07
*RAD51*	1.02	0.05	*STAT3*	−1.10	0.04	*XRCC3*	1.04	0.31
*RAD9A*	1.04	0.04	*STAT5B*	−1.08	0.09	*XRCC5*	1.10	0.11
*RASA1*	−1.24	0.05	*TLR1*	1.15	0.03	*XRCC6BP1*	−1.20	0.10

**Table A4:** TNF-α induced gene expression in HEK shRNA RelA cells (RelA k.d.) compared to the original cell line HEK-pNF-κB-d2EGFP/Neo L2 (HEK wt) using the RT2 Profiler PCR array.

	HEK wt		RelA k.d.	
Symbol	µ	SE	µ	SE
*ACTB*	−1.19	0.05	−1.26	0.07
*ADM*	−1.88	0.08	1.39	0.27
*AGT*	1.57	0.42	−1.06	0.11
*AKT1*	−1.36	0.08	1.04	0.16
*ALDH3A2*	−1.19	0.02	−1.23	0.03
*ATM*	−1.01	0.03	1.19	0.06
*ATR*	−1.50	0.05	1.35	0.03
*B2M*	1.58	0.14	1.27	0.07
*BCL2A1*	2.22	0.32	1.47	0.20
*BCL2L1*	1.65	0.19	1.17	0.08
*BCL3*	2.38	0.44	1.67	0.47
*BIRC2*	1.54	0.13	1.27	0.12
*BIRC3*	4.76	0.98	2.03	0.47
*BIRC5*	−1.36	0.10	−1.09	0.01
*BRCA2*	1.21	0.07	1.48	0.22
*BRCA5*	2.04	0.67	1.47	0.33
*C3*	5.97	1.39	1.97	0.57
*CASP3*	−1.04	0.10	1.44	0.02
*CASP8*	1.97	0.74	1.80	0.56
*CASP9*	1.08	0.07	−1.02	0.05
*CAT*	−1.44	0.18	−1.18	0.11
*CCL11*	3.73	1.51	1.84	0.57
*CCL2*	3.04	0.24	−1.28	0.09
*CCL22*	1.39	0.24	−1.15	0.04
*CCL5*	4.79	1.79	29.02	12.66
*CCND1*	−2.35	0.11	−1.55	0.09
*CCNE1*	−1.13	0.09	−1.10	0.04
*CCNE2*	1.40	0.33	1.56	0.22
*CCR5*	1.61	0.49	−2.87	0.06
*CD40*	2.83	0.53	1.51	0.30
*CD69*	3.37	0.23	−1.15	0.04
*CD80*	2.06	0.50	2.32	0.77
*CD83*	5.68	0.82	1.67	0.08
*CDC2*	1.16	0.16	1.38	0.31
*CDC25A*	1.25	0.02	−1.04	0.11
*CDKN1A*	2.85	0.50	2.59	0.59
*CDKN2A*	−1.35	0.04	−1.14	0.04
*CFB*	2.21	0.36	1.36	0.17
*CHEK1*	1.35	0.25	1.15	0.15
*CHEK2*	1.18	0.06	1.14	0.09
*CSF1*	2.55	0.25	−1.14	0.02
*CSF2*	1.16	0.37	−1.32	0.10
*CSF2RB*	−1.07	0.05	−1.15	0.04
*CSF3*	1.07	0.30	1.13	0.21
*CUL1*	1.08	0.15	−1.09	0.06
*CXCL1*	47.46	11.75	−1.05	0.08
*CXCL10*	23.38	5.72	−1.18	0.06
*CXCL2*	18.29	3.92	1.72	0.33
*CXCL9*	2.08	0.62	1.72	0.45
*DDB1*	−1.10	0.27	−1.33	0.10
*DDIT3*	1.88	0.63	1.91	0.50
*DNAJA1*	1.48	0.24	1.07	0.02
*E2F1*	−1.31	0.28	−1.28	0.18
*EGFR*	1.80	0.20	1.33	0.14
*EGR1*	−1.06	0.18	1.23	0.03
*EGR2*	1.98	0.57	1.81	0.47
*EIF2AK2*	1.06	0.06	1.09	0.04
*ERCC3*	1.26	0.01	1.12	0.17
*EXO1*	1.97	0.67	−4.29	0.02
*F3*	2.66	0.30	1.66	0.22
*F8*	−1.23	0.10	1.26	0.02
*FANCG*	1.26	0.08	1.18	0.05
*FAS*	−1.39	0.13	−1.13	0.16
*FASLG*	1.28	0.47	−1.01	0.18
*GADD45A*	1.38	0.15	1.86	0.00
*GADD45B*	2.01	0.56	2.60	0.91
*GADD45G*	−1.01	0.24	1.12	0.20
*GAPDH*	−1.29	0.06	−1.32	0.04
*GJA1*	−1.03	0.12	−1.46	0.17
*GPX1*	−1.17	0.13	−1.34	0.03
*GTF2H2*	1.42	0.02	1.24	0.13
*HPRT1*	−1.13	0.07	1.40	0.17
*HRK*	2.12	0.76	2.45	1.08
*HSF1*	1.60	0.44	−1.00	0.07
*HSP90AA2*	1.17	0.16	1.05	0.07
*HSPA1A*	−1.51	0.15	−1.04	0.08
*ICAM1*	2.12	0.36	−2.56	0.05
*IFNB1*	−1.01	0.05	−1.15	0.04
*IFNG*	−1.36	0.17	−1.15	0.04
*IKBKB*	1.20	0.14	1.05	0.00
*IKBKE*	1.22	0.08	−1.83	0.03
*IKBKG*	1.01	0.33	−1.75	0.06
*IL10*	1.41	0.26	−1.80	0.17
*IL12B*	1.82	0.32	1.42	0.07
*IL15*	2.83	0.63	1.59	0.07
*IL1A*	1.59	0.19	1.38	0.23
*IL1B*	−1.10	0.07	1.27	0.12
*IL1R2*	4.02	1.29	1.71	0.30
*IL1RN*	1.39	0.24	−1.15	0.04
*IL2*	−1.27	0.13	−1.15	0.04
*IL2RA*	1.35	0.21	−1.15	0.04
*IL4*	1.62	0.43	−1.12	0.14
*IL6*	1.01	0.23	1.05	0.18
*IL6*	1.86	0.58	2.39	0.50
*IL8*	17.83	5.19	−1.15	0.04
*INS*	−1.27	0.12	1.29	0.11
*IRF1*	3.16	0.40	−1.18	0.12
*JUN*	1.39	0.69	1.15	0.01
*KRAS*	1.11	0.02	−1.42	0.10
*LIG1*	−1.24	0.29	1.58	0.38
*LTA*	1.52	0.51	2.06	0.67
*LTB*	1.92	0.58	−1.80	0.13
*MAP2K6*	−1.57	0.12	−1.08	0.11
*MAPK1*	−1.13	0.13	1.02	0.13
*MDM2*	−1.01	0.13	2.01	0.37
*MMP9*	3.34	0.61	−1.53	0.06
*MT2A*	−1.13	0.02	−1.04	0.45
*MT3*	1.86	0.58	−1.10	0.16
*MYC*	−1.84	0.08	2.09	0.70
*MYD88*	1.49	0.35	1.01	0.29
*NBN*	−1.18	0.06	1.12	0.08
*NCOA3*	−1.02	0.06	1.22	0.04
*NFKB1*	2.88	0.28	1.47	0.13
*NFKB2*	2.90	0.61	1.79	0.40
*NFKBIA*	11.88	1.66	2.52	0.30
*NOS2*	1.91	0.52	1.32	0.20
*NQO1*	−1.76	0.10	−1.10	0.05
*NR4A2*	1.17	0.18	1.22	0.21
*OGG1*	−1.19	0.10	−1.19	0.19
*P53AIP1*	1.64	0.09	1.42	0.41
*PCNA*	1.36	0.14	1.25	0.18
*PDGFB*	1.17	0.12	−1.15	0.04
*PIK3Ca*	1.20	0.08	1.19	0.06
*PLAU*	3.35	0.57	2.64	0.58
*PRKCA*	−1.00	0.17	−1.21	0.05
*PTGS2*	3.99	0.53	2.17	0.39
*RAD51*	−1.00	0.02	1.00	0.07
*RAD9A*	1.10	0.02	−1.31	0.04
*RASA1*	1.11	0.07	1.27	0.07
*RB1*	1.07	0.03	1.08	0.05
*REL*	2.40	0.25	1.33	0.07
*RELA*	1.69	0.17	−1.11	0.06
*RELB*	1.82	0.55	1.21	0.23
*RPL13A*	−1.29	0.09	−1.12	0.15
*RPLP0*	1.46	0.12	1.10	0.05
*SELE*	1.86	0.71	1.06	0.19
*SELP*	1.17	0.11	−1.15	0.04
*SNAP25*	1.48	0.31	2.06	0.64
*SOD1*	−1.28	0.13	−1.16	0.05
*SOD2*	−1.17	0.09	−1.10	0.06
*SOD3*	1.86	0.58	−1.42	0.27
*SOS1*	1.25	0.19	1.08	0.14
*STAT1*	1.07	0.11	1.01	0.04
*STAT3*	−1.15	0.09	1.02	0.06
*STAT5B*	1.35	0.18	1.28	0.17
*TLR1*	−1.40	0.01	−2.09	0.02
*TNF*	60.95	12.42	3.40	0.54
*TNFRSF1B*	1.49	0.34	4.13	2.29
*TNFSF10*	1.73	0.37	2.07	0.78
*TP53*	1.17	0.05	1.16	0.06
*TP53BP2*	1.31	0.17	−1.00	0.09
*TP73*	1.11	0.12	−2.03	0.04
*TRADD*	1.09	0.06	−1.42	0.04
*TRAF2*	1.23	0.11	1.04	0.05
*UNG*	−1.23	0.12	−1.09	0.05
*VCAM1*	−1.36	0.15	−1.15	0.04
*WT1*	1.20	0.01	−1.64	0.13
*XIAP*	1.50	0.21	1.37	0.17
*XRCC1*	1.04	0.13	−1.17	0.02
*XRCC2*	1.17	0.14	1.42	0.11
*XRCC3*	−1.28	0.27	−1.65	0.13
*XRCC5*	−1.17	0.12	1.05	0.01
*XRCC6BP1*	−1.08	0.02	−1.09	0.15

**Table A5:** Relative gene expression in HEK-pNF-κB-d2EGFP/Neo L2 (HEK wt) and HEK shRNA RelA (RelA k.d.) cells after irradiation using the RT2 Profiler PCR array: X-rays.

	HEK wt						RelA k.d.					
	0.5 Gy		4 Gy		8 Gy		0.5 Gy		4 Gy		8 Gy	
Symbol	µ	SE	µ	SE	µ	SE	µ	SE	µ	SE	µ	SE
*ADM*	−1.09	0.11	−1.04	0.09	−1.16	0.08	1.02	0.20	1.18	0.23	1.15	0.22
*AGT*	−1.35	0.23	1.80	0.57	1.56	0.47	1.07	0.12	1.17	0.13	−1.00	0.11
*AKT1*	1.08	0.06	1.05	0.05	1.12	0.05	1.22	0.19	1.31	0.20	1.29	0.20
*ALDH3A2*	1.16	0.04	1.01	0.07	−1.08	0.06	1.06	0.04	−1.03	0.04	−1.01	0.04
*BCL2A1*	1.15	0.19	1.31	0.15	−1.15	0.12	−1.02	0.13	1.45	0.19	1.04	0.14
*BCL2L1*	1.01	0.11	−1.18	0.09	−1.12	0.13	−1.41	0.05	1.21	0.09	−1.24	0.06
*BIRC2*	−1.17	0.06	−1.14	0.04	1.02	0.07	−1.37	0.07	−1.02	0.09	−1.08	0.08
*BIRC3*	−1.31	0.11	1.96	0.38	2.53	0.29	−1.86	0.12	−1.02	0.23	−1.22	0.19
*C3*	1.98	0.32	1.98	0.32	1.05	0.14	−1.72	0.17	1.43	0.42	1.20	0.35
*CCL11*	1.40	0.57	2.66	0.78	2.63	0.77	1.88	0.58	1.90	0.59	1.23	0.38
*CCL2*	−1.21	0.08	1.24	0.15	2.25	0.20	1.25	0.15	1.10	0.13	1.04	0.12
*CCL22*	1.73	0.32	1.14	0.31	1.08	0.24	1.08	0.05	1.17	0.06	1.15	0.05
*CCL5*	−1.42	0.24	3.68	1.53	3.34	1.40	1.55	0.68	2.07	0.90	−1.47	0.30
*CCND1*	1.02	0.19	1.15	0.18	1.20	0.18	1.46	0.21	2.16	0.30	2.13	0.30
*CCR5*	−1.34	0.28	1.78	0.61	1.49	0.44	1.55	0.28	1.52	0.27	−1.01	0.18
*CD40*	1.09	0.18	1.27	0.15	1.21	0.16	−1.02	0.19	1.42	0.28	−1.14	0.17
*CD69*	1.28	0.10	1.14	0.11	−1.01	0.08	1.08	0.05	1.17	0.06	1.15	0.05
*CD80*	−1.63	0.17	1.45	0.35	1.29	0.25	−1.34	0.25	2.42	0.80	−1.35	0.24
*CD83*	−1.01	0.03	2.47	0.08	3.75	0.18	1.12	0.06	1.67	0.08	2.27	0.11
*CDKN1A*	1.41	0.19	1.81	0.19	1.84	0.21	1.17	0.33	1.58	0.45	2.04	0.58
*CFB*	1.21	0.14	−1.09	0.08	−1.20	0.09	−1.33	0.09	−1.39	0.09	−1.15	0.11
*CSF1*	1.31	0.12	1.52	0.13	1.92	0.14	1.13	0.02	1.28	0.03	1.30	0.03
*CSF2*	−1.49	0.27	−1.02	0.32	1.30	0.46	1.44	0.19	1.41	0.19	−1.06	0.13
*CSF2RB*	1.07	0.08	−1.00	0.08	−1.13	0.06	1.08	0.05	1.17	0.06	1.15	0.05
*CSF3*	−1.55	0.22	1.53	0.54	1.82	0.62	1.26	0.23	1.03	0.19	−1.31	0.14
*CXCL1*	1.04	0.20	3.57	0.56	6.96	1.01	1.06	0.09	1.52	0.12	2.02	0.16
*CXCL10*	−1.10	0.16	4.68	0.60	14.58	2.57	1.05	0.08	2.23	0.16	2.04	0.15
*CXCL2*	−1.35	0.13	2.33	0.33	3.01	0.40	−1.04	0.19	1.55	0.30	1.29	0.25
*CXCL9*	−2.11	0.17	1.58	0.56	1.75	0.48	−1.43	0.18	1.38	0.36	−1.25	0.21
*EGFR*	1.25	0.12	1.43	0.11	1.57	0.13	−1.10	0.10	1.04	0.11	−1.07	0.10
*EGR2*	−1.77	0.19	1.03	0.24	1.32	0.31	−1.65	0.16	1.95	0.51	−1.02	0.26
*F3*	−1.05	0.11	1.35	0.11	1.56	0.13	1.13	0.15	1.31	0.17	1.67	0.22
*F8*	−1.39	0.10	−1.36	0.08	−1.45	0.08	−1.35	0.01	−1.55	0.01	−1.48	0.01
*FAS*	1.35	0.30	1.31	0.21	1.57	0.25	1.68	0.31	1.85	0.34	1.86	0.34
*FASLG*	−1.78	0.25	1.81	0.77	2.39	0.97	1.28	0.23	1.40	0.25	−1.61	0.11
*GADD45B*	−1.83	0.17	1.66	0.49	1.50	0.37	−1.30	0.27	−1.43	0.24	−1.60	0.22
*ICAM1*	1.46	0.24	1.29	0.17	1.03	0.16	−1.02	0.12	−1.00	0.12	1.11	0.14
*IFNB1*	1.12	0.07	1.27	0.06	−1.27	0.09	1.08	0.05	1.17	0.06	1.15	0.05
*IFNG*	−1.32	0.22	1.44	0.39	1.41	0.41	1.08	0.05	1.17	0.06	1.15	0.05
*IL12B*	−1.36	0.16	1.45	0.33	1.64	0.31	−1.02	0.05	2.01	0.10	1.13	0.05
*IL15*	1.14	0.23	2.22	0.58	2.31	0.49	1.08	0.05	1.46	0.06	1.55	0.07
*IL1A*	−1.52	0.11	−1.33	0.11	−1.31	0.09	−1.70	0.12	1.26	0.27	−1.24	0.17
*IL1B*	1.03	0.09	−1.12	0.10	−1.36	0.09	1.02	0.10	1.10	0.11	1.08	0.10
*IL1R2*	−1.80	0.16	−1.00	0.24	−1.29	0.18	−1.27	0.14	1.41	0.25	−1.13	0.16
*IL1RN*	1.73	0.32	1.14	0.31	1.08	0.24	1.08	0.05	1.17	0.06	1.15	0.05
*IL2*	−1.16	0.18	1.14	0.19	1.03	0.18	1.08	0.05	1.17	0.06	1.15	0.05
*IL2RA*	1.67	0.28	1.21	0.27	1.30	0.18	1.08	0.05	1.17	0.06	1.15	0.05
*IL4*	−1.36	0.23	1.51	0.46	1.20	0.33	1.10	0.18	−1.17	0.14	−1.60	0.10
*IL6*	−1.30	0.22	1.75	0.51	1.53	0.45	1.19	0.12	1.09	0.11	1.07	0.11
*IL8*	−1.73	0.19	2.42	0.66	3.74	0.86	1.20	0.19	1.38	0.22	1.01	0.16
*INS*	−1.16	0.17	1.22	0.20	1.48	0.34	1.08	0.05	1.17	0.06	1.15	0.05
*IRF1*	1.27	0.14	1.45	0.15	2.19	0.25	1.08	0.09	1.41	0.12	1.56	0.13
*LTA*	−1.19	0.35	1.77	0.64	1.89	0.60	−1.29	0.19	1.86	0.45	1.00	0.24
*LTB*	−1.87	0.20	1.23	0.38	1.13	0.30	−1.54	0.21	2.67	0.87	1.30	0.42
*MAP2K6*	−1.20	0.18	−1.91	0.10	−2.78	0.06	−1.15	0.21	−1.09	0.22	−1.02	0.24
*MMP9*	−1.37	0.04	−1.36	0.03	−1.48	0.03	−1.42	0.13	−1.28	0.14	−1.18	0.16
*MYC*	1.03	0.05	−1.02	0.06	−1.22	0.04	−1.19	0.15	−1.24	0.14	−1.24	0.14
*MYD88*	−1.89	0.14	1.24	0.36	1.13	0.27	1.57	0.53	1.60	0.54	1.41	0.47
*NCOA3*	1.04	0.08	−1.15	0.05	−1.20	0.05	−1.13	0.06	−1.11	0.06	−1.33	0.05
*NFKB1*	−1.08	0.13	1.62	0.17	2.73	0.30	1.17	0.03	1.21	0.04	1.31	0.04
*NFKB2*	1.01	0.12	2.93	0.33	4.07	0.45	1.09	0.18	1.48	0.24	1.62	0.26
*NFKBIA*	−1.07	0.08	2.65	0.34	4.26	0.40	−1.13	0.10	1.47	0.17	1.69	0.19
*NQO1*	1.10	0.12	−1.10	0.12	−1.17	0.10	1.18	0.06	1.21	0.06	1.49	0.08
*NR4A2*	1.43	0.25	−1.10	0.19	1.03	0.23	−1.02	0.17	1.08	0.19	1.73	0.30
*PDGFB*	1.41	0.16	1.13	0.17	−1.02	0.14	1.08	0.05	1.17	0.06	1.15	0.05
*PLAU*	−1.11	0.15	2.09	0.49	2.18	0.41	1.22	0.27	1.26	0.28	−1.29	0.17
*PTGS2*	−1.03	0.03	1.41	0.03	1.77	0.05	−1.62	0.11	−1.21	0.15	−1.35	0.13
*REL*	1.10	0.15	1.02	0.14	1.40	0.17	−1.32	0.04	−1.07	0.05	−1.07	0.05
*RELA*	1.10	0.06	1.07	0.04	1.19	0.05	1.00	0.06	1.14	0.07	1.05	0.06
*RELB*	−1.73	0.21	1.68	0.60	1.87	0.51	−1.13	0.17	1.09	0.20	1.02	0.19
*SELE*	−1.79	0.25	1.70	0.68	1.92	0.71	1.36	0.24	1.32	0.24	−1.41	0.13
*SELP*	1.41	0.15	−1.01	0.21	−1.08	0.15	1.08	0.05	1.17	0.06	1.15	0.05
*SNAP25*	1.05	0.26	1.21	0.22	1.06	0.19	1.59	0.49	1.86	0.58	1.96	0.61
*SOD2*	−1.05	0.07	−1.08	0.05	−1.00	0.07	−1.03	0.09	1.02	0.10	1.15	0.11
*STAT1*	1.15	0.14	−1.17	0.09	−1.10	0.10	−1.08	0.04	−1.14	0.04	−1.25	0.03
*STAT3*	−1.14	0.10	1.03	0.11	1.12	0.13	−1.13	0.06	−1.15	0.05	−1.16	0.05
*STAT5B*	1.08	0.16	1.33	0.19	1.24	0.15	1.00	0.13	−1.08	0.12	−1.17	0.11
*TNF*	1.01	0.11	5.10	0.99	12.90	1.72	1.08	0.05	1.17	0.06	1.15	0.05
*TNFRSF1B*	−1.81	0.16	1.02	0.22	1.11	0.22	−2.15	0.26	4.10	2.28	2.39	1.33
*TNFSF10*	−1.48	0.17	1.11	0.21	−1.02	0.18	−1.61	0.23	2.81	1.06	1.64	0.62
*TP53*	1.13	0.02	1.39	0.04	1.67	0.08	−1.14	0.02	−1.02	0.02	−1.04	0.02
*TRAF2*	1.06	0.11	1.04	0.09	1.12	0.10	1.10	0.05	−1.10	0.04	−1.03	0.04
*VCAM1*	−1.27	0.20	4.24	1.56	2.94	0.87	1.08	0.05	1.17	0.06	1.15	0.05
*XIAP*	−1.07	0.11	−1.18	0.09	−1.23	0.07	−1.13	0.11	−1.17	0.10	−1.27	0.10
*ACTB*	1.12	0.10	−1.16	0.08	−1.08	0.07	1.21	0.17	1.08	0.16	−1.04	0.14
*B2M*	−1.05	0.07	1.29	0.14	1.29	0.09	−1.09	0.04	−1.06	0.04	1.08	0.04
*GAPDH*	−1.14	0.04	−1.03	0.04	−1.10	0.05	1.03	0.05	−1.06	0.05	−1.17	0.04
*HPRT1*	1.04	0.04	1.01	0.03	−1.02	0.04	−1.08	0.12	1.10	0.14	1.21	0.16
*RPLP0*	1.06	0.05	1.03	0.07	1.01	0.05	1.01	0.05	1.00	0.05	−1.01	0.05

**Table A6:** Relative gene expression in HEK-pNF-κB-d2EGFP/Neo L2 (HEK wt) and HEK shRNA RelA (RelA k.d.) cells after irradiation using the RT2 Profiler PCR array: Ti ions.

	HEK wt				RelA k.d.			
	0.5 Gy		4 Gy		0.5 Gy		4 Gy	
Symbol	µ	SE	µ	SE	µ	SE	µ	SE
*ADM*	−1.44	0.08	1.14	0.13	1.22	0.24	1.24	0.24
*AGT*	−1.88	0.16	−1.48	0.21	−1.36	0.08	1.11	0.13
*AKT1*	−1.01	0.05	1.11	0.06	−1.11	0.14	−1.16	0.13
*ALDH3A2*	−1.05	0.03	−1.16	0.03	1.08	0.04	−1.15	0.04
*BCL2A1*	−1.21	0.13	1.04	0.17	1.12	0.15	−1.23	0.11
*BCL2L1*	1.17	0.13	−1.16	0.10	1.36	0.10	−1.68	0.04
*BIRC2*	1.08	0.07	1.06	0.07	1.26	0.12	−1.17	0.08
*BIRC3*	1.34	0.20	1.66	0.24	1.29	0.30	−1.16	0.20
*C3*	1.58	0.25	1.03	0.16	−1.46	0.20	−1.99	0.15
*CCL11*	1.97	0.81	1.10	0.45	1.46	0.45	1.68	0.52
*CCL2*	1.18	0.11	2.94	0.27	1.57	0.19	2.64	0.31
*CCL22*	1.24	0.23	1.57	0.29	−1.26	0.04	−1.12	0.04
*CCL5*	−1.97	0.17	−1.56	0.22	−1.89	0.23	1.22	0.53
*CCND1*	−1.77	0.11	1.03	0.19	−1.23	0.11	−1.24	0.11
*CCR5*	−1.02	0.37	−2.13	0.18	1.04	0.19	1.35	0.24
*CD40*	1.58	0.26	−1.17	0.14	1.15	0.23	−1.30	0.15
*CD69*	−1.09	0.07	1.16	0.09	−1.26	0.04	−1.12	0.04
*CD80*	−1.01	0.27	−1.55	0.18	2.03	0.67	−1.53	0.22
*CD83*	1.25	0.04	4.37	0.14	1.09	0.05	1.42	0.07
*CDKN1A*	−1.39	0.10	1.21	0.17	−1.35	0.21	−1.07	0.26
*CFB*	1.01	0.12	1.14	0.14	1.57	0.20	−1.01	0.12
*CSF1*	−1.15	0.08	1.69	0.15	−1.06	0.02	−1.01	0.02
*CSF2*	−1.82	0.22	−1.30	0.31	−3.46	0.04	1.48	0.20
*CSF2RB*	−1.30	0.06	−1.03	0.07	−1.26	0.04	−1.12	0.04
*CSF3*	−1.44	0.24	−1.44	0.24	1.02	0.19	1.46	0.27
*CXCL1*	1.01	0.19	7.55	1.44	−1.48	0.05	1.21	0.10
*CXCL10*	−1.27	0.14	6.70	1.20	−1.30	0.06	−1.15	0.06
*CXCL2*	1.30	0.22	3.48	0.59	1.11	0.21	1.00	0.19
*CXCL9*	1.02	0.37	−2.17	0.17	1.14	0.30	−1.66	0.16
*EGFR*	−1.33	0.07	−1.12	0.09	−1.05	0.10	−1.08	0.10
*EGR2*	1.17	0.39	−1.66	0.20	1.57	0.41	−1.52	0.17
*F3*	−1.15	0.10	1.47	0.17	−1.01	0.13	−1.00	0.13
*F8*	−1.22	0.12	−1.06	0.13	1.19	0.01	−1.01	0.01
*FAS*	−2.11	0.11	−1.61	0.14	1.14	0.21	1.15	0.21
*FASLG*	−1.40	0.32	−1.51	0.30	−1.10	0.16	1.15	0.21
*GADD45B*	−1.06	0.30	2.19	0.69	1.69	0.59	2.06	0.72
*ICAM1*	−1.57	0.10	−1.64	0.10	1.14	0.14	1.37	0.17
*IFNB1*	−1.24	0.05	1.02	0.06	−1.26	0.04	−1.12	0.04
*IFNG*	−1.84	0.16	−1.46	0.20	−1.26	0.04	−1.12	0.04
*IL12B*	−1.35	0.16	−1.49	0.14	1.25	0.06	1.30	0.06
*IL15*	−1.32	0.15	1.07	0.22	−1.21	0.04	−1.41	0.03
*IL1A*	−1.39	0.12	−1.83	0.09	1.57	0.33	−1.40	0.15
*IL1B*	−1.35	0.07	−1.07	0.09	−1.02	0.10	−1.18	0.08
*IL1R2*	−1.29	0.22	−1.99	0.14	1.01	0.18	−1.80	0.10
*IL1RN*	1.24	0.23	1.57	0.29	−1.26	0.04	−1.12	0.04
*IL2*	−1.62	0.13	−1.28	0.16	−1.26	0.04	−1.12	0.04
*IL2RA*	1.20	0.20	1.52	0.25	−1.26	0.04	−1.12	0.04
*IL4*	−1.12	0.28	−1.17	0.26	−1.26	0.13	1.02	0.16
*IL6*	−1.81	0.15	−1.43	0.20	−1.27	0.08	1.09	0.11
*IL8*	1.38	0.44	6.10	1.96	−1.26	0.13	1.44	0.23
*INS*	−1.62	0.12	−1.28	0.15	−1.26	0.04	−1.12	0.04
*IRF1*	1.06	0.12	2.85	0.33	−1.09	0.08	1.40	0.11
*LTA*	1.05	0.44	−2.07	0.20	1.28	0.31	−1.39	0.17
*LTB*	1.16	0.44	−2.18	0.17	1.91	0.62	−1.49	0.22
*MAP2K6*	−1.17	0.19	−1.63	0.13	1.99	0.48	1.44	0.35
*MMP9*	1.24	0.07	−1.04	0.06	1.09	0.20	1.07	0.20
*MYC*	−1.14	0.04	1.21	0.05	1.67	0.29	1.97	0.35
*MYD88*	−1.05	0.24	−1.25	0.21	−1.35	0.25	−1.10	0.30
*NCOA3*	−1.18	0.06	1.06	0.08	1.18	0.08	−1.17	0.06
*NFKB1*	−1.24	0.12	2.09	0.30	1.04	0.03	1.05	0.03
*NFKB2*	−1.25	0.09	2.52	0.29	−1.02	0.16	1.04	0.17
*NFKBIA*	−1.11	0.08	3.55	0.31	1.05	0.12	1.47	0.17
*NQO1*	−1.27	0.09	−1.34	0.08	1.13	0.06	−1.14	0.04
*NR4A2*	1.09	0.19	1.11	0.19	−1.20	0.14	−1.40	0.12
*PDGFB*	1.02	0.11	1.28	0.14	−1.26	0.04	−1.12	0.04
*PLAU*	−1.64	0.10	1.08	0.18	−1.09	0.20	−1.47	0.15
*PTGS2*	1.39	0.04	2.12	0.06	1.07	0.19	1.01	0.18
*REL*	1.11	0.15	1.84	0.25	1.02	0.05	−1.17	0.04
*RELA*	1.17	0.07	1.48	0.08	1.21	0.07	1.15	0.07
*RELB*	−1.39	0.26	−1.09	0.33	1.01	0.19	−1.20	0.16
*SELE*	−1.09	0.41	−1.34	0.33	−1.00	0.18	1.06	0.19
*SELP*	1.01	0.11	1.28	0.13	−1.26	0.04	−1.12	0.04
*SNAP25*	−1.74	0.14	−2.36	0.10	−1.32	0.24	−1.69	0.18
*SOD2*	−1.17	0.06	−1.06	0.06	1.24	0.12	1.29	0.13
*STAT1*	1.19	0.14	1.40	0.17	−1.01	0.04	−1.03	0.04
*STAT3*	−1.29	0.09	−1.32	0.09	1.11	0.07	−1.08	0.06
*STAT5B*	−1.16	0.13	−1.18	0.13	−1.14	0.12	−1.40	0.10
*TNF*	1.08	0.12	6.72	0.76	−1.26	0.04	−1.12	0.04
*TNFRSF1B*	1.07	0.30	−2.04	0.14	2.88	1.60	−2.65	0.21
*TNFSF10*	1.06	0.27	−1.65	0.15	1.58	0.60	−2.19	0.17
*TP53*	−1.03	0.02	1.49	0.03	−1.12	0.02	−1.03	0.02
*TRAF2*	−1.13	0.09	1.32	0.14	−1.00	0.04	1.15	0.05
*VCAM1*	−1.77	0.15	−1.10	0.24	−1.26	0.04	−1.12	0.04
*XIAP*	−1.01	0.12	1.19	0.14	−1.02	0.12	−1.32	0.09
*ACTB*	1.25	0.11	1.12	0.10	1.19	0.17	1.24	0.18
*B2M*	−1.08	0.07	−1.01	0.08	−1.06	0.04	−1.07	0.04
*GAPDH*	−1.02	0.05	−1.12	0.04	1.13	0.05	1.10	0.05
*HPRT1*	−1.10	0.04	−1.05	0.04	−1.21	0.11	−1.21	0.11
*RPLP0*	1.01	0.05	1.10	0.05	1.01	0.05	1.01	0.05

## References

[1] Berger T., Bilski P., Hajek M., Puchalska M., Reitz G. (2013). The MATROSHKA Experiment: Results and Comparison from Extravehicular Activity (MTR-1) and Intravehicular Activity (MTR-2A/2B) Exposure. Radiat. Res..

[2] Puchalska M., Bilski P., Berger T., Hajek M., Horwacik T., Korner C., Olko P., Shurshakov V., Reitz G. (2014). NUNDO: A Numerical Model of a Human Torso Phantom and Its Application to Effective Dose Equivalent Calculations for Astronauts at the ISS. Radiat. Environ. Biophys..

[3] Zeitlin C., Hassler D.M., Cucinotta F.A., Ehresmann B., Wimmer-Schweingruber R.F., Brinza D.E., Kang S., Weigle G., Bottcher S., Bohm E. (2013). Measurements of Energetic Particle Radiation in Transit to Mars on the Mars Science Laboratory. Science.

[4] Hassler D.M., Zeitlin C., Wimmer-Schweingruber R.F., Ehresmann B., Rafkin S., Eigenbrode J.L., Brinza D.E., Weigle G., Bottcher S., Bohm E. (2014). Mars’ Surface Radiation Environment Measured with the Mars Science Laboratory’s Curiosity Rover. Science.

[5] National Council on Radiation Protection and Measurements (2006). Information Needed to Make Radiation Protection Recommendations for Space Missions beyond Low-Earth Orbit: Recommendations of the National Council on Radiation Protection and Measurements.

[6] Cucinotta F.A., Manuel F.K., Jones J., Iszard G., Murrey J., Djojonegro B., Wear M. (2001). Space Radiation and Cataracts in Astronauts. Radiat. Res..

[7] Chylack L.T., Peterson L.E., Feiveson A.H., Wear M.L., Manuel F.K., Tung W.H., Hardy D.S., Marak L.J., Cucinotta F.A. (2009). NASA Study of Cataract in Astronauts (NASCA). Report 1: Cross-Sectional Study of the Relationship of Exposure to Space Radiation and Risk of Lens Opacity. Radiat. Res..

[8] Hughson R.L., Helm A., Durante M. (2018). Heart in Space: Effect of the Extraterrestrial Environment on the Cardiovascular System. Nat. Rev. Cardiol..

[9] Jandial R., Hoshide R., Waters J.D., Limoli C.L. (2018). Space-Brain: The Negative Effects of Space Exposure on the Central Nervous System. Surg. Neurol. Intern..

[10] Sanzari J.K., Wan X.S., Muehlmatt A., Lin L., Kennedy A.R. (2015). Comparison of Changes over Time in Leukocyte Counts in Yucatan Minipigs Irradiated with Simulated Solar Particle Event-Like Radiation. Life Sci. Space Res..

[11] Sanzari J.K., Diffenderfer E.S., Hagan S., Billings P.C., Gridley D.S., Seykora J.T., Kennedy A.R., Cengel K.A. (2015). Dermatopathology Effects of Simulated Solar Particle Event Radiation Exposure in the Porcine Model. Life Sci. Space Res..

[12] Pecaut M.J., Gridley D.S. (2010). The Impact of Mouse Strain on Iron Ion Radio-Immune Response of Leukocyte Populations. Int. J. Radiat. Biol..

[13] Georgakilas A.G., O’Neill P., Stewart R.D. (2013). Induction and Repair of Clustered DNA Lesions: What do We Know So Far?. Radiat. Res..

[14] Hellweg C.E., Spitta L.F., Henschenmacher B., Diegeler S., Baumstark-Khan C. (2016). Transcription Factors in the Cellular Response to Charged Particle Exposure. Front. Oncol..

[15] Colombo F., Zambrano S., Agresti A. (2018). NF-kappaB, the Importance of Being Dynamic: Role and Insights in Cancer. Biomedicines.

[16] Brach M.A., Hass R., Sherman M.L., Gunji H., Weichselbaum R., Kufe D. (1991). Ionizing Radiation Induces Expression and Binding Activity of the Nuclear Factor Kappa B. J. Clin. Investig..

[17] Sen R., Baltimore D. (1986). Inducibility of Kappa Immunoglobulin Enhancer-Binding Protein Nf-Kappa B by a Posttranslational Mechanism. Cell.

[18] Baumstark-Khan C., Hellweg C.E., Arenz A., Meier M.M. (2005). Cellular Monitoring of the Nuclear Factor kappaB Pathway for Assessment of Space Environmental Radiation. Radiat. Res..

[19] Hellweg C.E., Baumstark-Khan C., Schmitz C., Lau P., Meier M.M., Testard I., Berger T., Reitz G. (2011). Carbon-Ion-Induced Activation of the NF-kappaB Pathway. Radiat. Res..

[20] Hellweg C.E., Baumstark-Khan C., Schmitz C., Lau P., Meier M.M., Testard I., Berger T., Reitz G. (2011). Activation of the Nuclear Factor kappaB Pathway by Heavy Ion Beams of Different Linear Energy Transfer. Int. J. Radiat. Biol..

[21] Reitz G., Berger T., Bilski P., Facius R., Hajek M., Petrov V., Puchalska M., Zhou D., Bossler J., Akatov Y. (2009). Astronaut’s Organ Doses Inferred from Measurements in a Human Phantom Outside the International Space Station. Radiat. Res..

[22] Habraken Y., Piette J. (2006). NF-kappaB Activation by Double-Strand Breaks. Biochem. Pharmacol..

[23] Hellweg C.E. (2015). The Nuclear Factor kappaB Pathway: A link to the Immune System in the Radiation Response. Cancer Lett..

[24] Ghosh S., May M.J., Kopp E.B. (1998). NF-kappa B and Rel Proteins: Evolutionarily Conserved Mediators of Immune Responses. Annu. Rev. Immunol..

[25] Baichwal V.R., Baeuerle P.A. (1997). Activate NF-kappa B or Die?. Curr. Biol..

[26] Chishti A.A., Baumstark-Khan C., Koch K., Kolanus W., Feles S., Konda B., Azhar A., Spitta L.F., Henschenmacher B., Diegeler S. (2018). Linear Energy Transfer Modulates Radiation-Induced NF-kappa B Activation and Expression of its Downstream Target Genes. Radiat. Res..

[27] Hellweg C.E., Baumstark-Khan C., Horneck G. (2003). Generation of Stably Transfected Mammalian Cell Lines as Fluorescent Screening Assay for NF-kappaB Activation-Dependent Gene Expression. J. Biomol. Screen..

[28] Li X., Zhao X., Fang Y., Jiang X., Duong T., Fan C., Huang C.C., Kain S.R. (1998). Generation of Destabilized Green Fluorescent Protein as a Transcription Reporter. J. Biol. Chem..

[29] Natarajan M., Aravindan N., Meltz M.L., Herman T.S. (2002). Post-Translational Modification of I-kappa B Alpha Activates NF-kappa B in Human Monocytes Exposed to 56Fe Ions. Radiat. Environ. Biophys..

[30] Tungjai M., Whorton E.B., Rithidech K.N. (2013). Persistence of Apoptosis and Inflammatory Responses in the Heart and Bone Marrow of Mice Following Whole-Body Exposure to (2)(8)Silicon ((2)(8)Si) ions. Radiat. Environ. Biophys..

[31] Hickson I., Zhao Y., Richardson C.J., Green S.J., Martin N.M., Orr A.I., Reaper P.M., Jackson S.P., Curtin N.J., Smith G.C. (2004). Identification and Characterization of a Novel and Specific Inhibitor of the Ataxia-Telangiectasia Mutated Kinase ATM. Cancer Res..

[32] Xue L., Yu D., Furusawa Y., Okayasu R., Tong J., Cao J., Fan S. (2009). Regulation of ATM in DNA Double Strand Break Repair Accounts for the Radiosensitivity in Human Cells Exposed to High Linear Energy Transfer Ionizing Radiation. Mutat. Res..

[33] Takahashi A., Yamakawa N., Kirita T., Omori K., Ishioka N., Furusawa Y., Mori E., Ohnishi K., Ohnishi T. (2008). DNA Damage Recognition Proteins Localize along Heavy Ion Induced Tracks in the Cell Nucleus. J. Radiat. Res..

[34] Ghosh S., Narang H., Sarma A., Krishna M. (2011). DNA Damage Response Signaling in Lung Adenocarcinoma A549 Cells Following Gamma and Carbon Beam Irradiation. Mutat. Res..

[35] Li Q., Gao Y., Xu Z.G., Jiang H., Yu Y.Y., Zhu Z.G. (2013). Effect of Antisense Oligodeoxynucleotide Targeted Against NF-kappaB/P65 on Cell Proliferation and Tumorigenesis of Gastric Cancer. Clin. Exp. Med..

[36] Wang F., He W., Fanghui P., Wang L., Fan Q. (2013). NF-kappaBP65 Promotes Invasion and Metastasis of Oesophageal Squamous Cell Cancer by Regulating Matrix Metalloproteinase-9 and Epithelial-to-Mesenchymal Transition. Cell Biol. Int..

[37] Bonavia R., Inda M.M., Vandenberg S., Cheng S.Y., Nagane M., Hadwiger P., Tan P., Sah D.W., Cavenee W.K., Furnari F.B. (2012). EGFRvIII Promotes Glioma Angiogenesis and Growth through the NF-kappaB, Interleukin-8 Pathway. Oncogene.

[38] Shi Y., Wang S.Y., Yao M., Sai W.L., Wu W., Yang J.L., Cai Y., Zheng W.J., Yao D.F. (2015). Chemosensitization of HepG2 Cells by Suppression of NF-kappaB/p65 Gene Transcription with Specific-siRNA. World J. Gastroenterol..

[39] Xiao J., Duan X., Yin Q., Miao Z., Yu H., Chen C., Zhang Z., Wang J., Li Y. (2013). The Inhibition of Metastasis and Growth of Breast Cancer by Blocking the NF-kappaB Signaling Pathway Using Bioreducible PEI-based/p65 shRNA Complex Nanoparticles. Biomaterials.

[40] Toualbi-Abed K., Daniel F., Guller M.C., Legrand A., Mauriz J.L., Mauviel A., Bernuau D. (2008). Jun D Cooperates with p65 to Activate the Proximal kappaB Site of the Cyclin D1 Promoter: Role of PI3K/PDK-1. Carcinogenesis.

[41] Widera D., Mikenberg I., Elvers M., Kaltschmidt C., Kaltschmidt B. (2006). Tumor Necrosis Factor Alpha Triggers Proliferation of Adult Neural Stem Cells via IKK/NF-kappaB Signaling. BMC Neurosci..

[42] Guttridge D.C., Albanese C., Reuther J.Y., Pestell R.G., Baldwin A.S. (1999). NF-kappaB Controls Cell Growth and Differentiation through Transcriptional Regulation of Cyclin D1. Mol. Cell. Biol..

[43] Galardi S., Mercatelli N., Farace M.G., Ciafre S.A. (2011). NF-kB and c-Jun Induce the Expression of the Oncogenic miR-221 and miR-222 in Prostate Carcinoma and Glioblastoma Cells. Nucleic Acids Res..

[44] Williams J.R., Zhang Y., Zhou H., Osman M., Cha D., Kavet R., Cuccinotta F., Dicello J.F., Dillehay L.E. (1999). Predicting Cancer Rates in Astronauts from Animal Carcinogenesis Studies and Cellular Markers. Mutat. Res..

[45] Watson C., Miller D.A., Chin-Sinex H., Losch A., Hughes W., Sweeney C., Mendonca M.S. (2009). Suppression of NF-kappaB Activity by Parthenolide Induces X-ray Sensitivity through Inhibition of Split-Dose Repair in TP53 Null Prostate Cancer Cells. Radiat. Res..

[46] Veuger S.J., Hunter J.E., Durkacz B.W. (2009). Ionizing Radiation-Induced NF-kappaB Activation Requires PARP-1 Function to Confer Radioresistance. Oncogene.

[47] Wang C.Y., Mayo M.W., Baldwin A.S. (1996). TNF- and Cancer Therapy-Induced Apoptosis: Potentiation by Inhibition of NF-kappaB. Science.

[48] Jung M., Dritschilo A. (2001). NF-kappa B Signaling Pathway as a Target for Human Tumor Radiosensitization. Semin. Radiat. Oncol..

[49] Russo S.M., Tepper J.E., Baldwin A.S., Liu R., Adams J., Elliott P., Cusack J.C. (2001). Enhancement of Radiosensitivity by Proteasome Inhibition: Implications for a Role of NF-kappaB. Int. J. Radiat. Oncol. Biol. Phys..

[50] Criswell T., Leskov K., Miyamoto S., Luo G., Boothman D.A. (2003). Transcription Factors Activated in Mammalian Cells after Clinically Relevant Doses of Ionizing Radiation. Oncogene.

[51] Thyss R., Virolle V., Imbert V., Peyron J.F., Aberdam D., Virolle T. (2005). NF-kappaB/Egr-1/Gadd45 are Sequentially Activated upon UVB Irradiation to Mediate Epidermal Cell Death. EMBO J..

[52] Kraft D., Rall M., Volcic M., Metzler E., Groo A., Stahl A., Bauer L., Nasonova E., Salles D., Taucher-Scholz G. (2015). NF-kappaB-dependent DNA Damage-Signaling Differentially Regulates DNA Double-Strand Break Repair Mechanisms in Immature and Mature Human Hematopoietic Cells. Leukemia.

[53] Mori E., Takahashi A., Yamakawa N., Kirita T., Ohnishi T. (2009). High LET Heavy Ion Radiation Induces p53-independent Apoptosis. J. Radiat. Res..

[54] Takahashi A., Matsumoto H., Yuki K., Yasumoto J., Kajiwara A., Aoki M., Furusawa Y., Ohnishi K., Ohnishi T. (2004). High-LET Radiation Enhanced Apoptosis but Not Necrosis Regardless of p53 Status. Int. J. Radiat. Oncol. Biol. Phys..

[55] Lu W., Zhang G., Zhang R., Flores L.G., Huang Q., Gelovani J.G., Li C. (2010). Tumor Site-Specific Silencing of NF-kappaB p65 by Targeted Hollow Gold Nanosphere-Mediated Photothermal Transfection. Cancer Res..

[56] Vlahopoulos S., Boldogh I., Casola A., Brasier A.R. (1999). Nuclear Factor-kappaB-dependent Induction of Interleukin-8 Gene Expression by Tumor Necrosis Factor Alpha: Evidence for an Antioxidant Sensitive Activating Pathway Distinct from Nuclear Translocation. Blood.

[57] Janus P., Szoltysek K., Zajac G., Stokowy T., Walaszczyk A., Widlak W., Wojtas B., Gielniewski B., Iwanaszko M., Braun R. (2018). Pro-Inflammatory Cytokine and High Doses of Ionizing Radiation have Similar Effects on the Expression of NF-kappaB-dependent Genes. Cell. Signal..

[58] Roach D.R., Bean A.G., Demangel C., France M.P., Briscoe H., Britton W.J. (2002). TNF Regulates Chemokine Induction Essential for Cell Recruitment, Granuloma Formation, and Clearance of Mycobacterial Infection. J. Immunol..

[59] Imadome K., Iwakawa M., Nojiri K., Tamaki T., Sakai M., Nakawatari M., Moritake T., Yanagisawa M., Nakamura E., Tsujii H. (2008). Upregulation of Stress-Response Genes with Cell Cycle Arrest Induced by Carbon Ion Irradiation in Multiple Murine Tumors Models. Cancer Biol. Ther..

[60] Coward W.R., Okayama Y., Sagara H., Wilson S.J., Holgate S.T., Church M.K. (2002). NF-kappa B and TNF-alpha: A Positive Autocrine Loop in Human Lung Mast Cells?. J. Immunol..

[61] May M.J., Ghosh S. (1998). Signal Transduction through NF-kappa B. Immunol. Today.

[62] Baldwin A.S. (1996). The NF-kappa B and I kappa B Proteins: New Discoveries and Insights. Annu. Rev. Immunol..

[63] Matsumoto Y., Iwakawa M., Furusawa Y., Ishikawa K., Aoki M., Imadome K., Matsumoto I., Tsujii H., Ando K., Imai T. (2008). Gene Expression Analysis in Human Malignant Melanoma Cell Lines Exposed to Carbon Beams. Int. J. Radiat. Biol..

[64] Souto-Carneiro M.M., Fritsch R., Sepulveda N., Lagareiro M.J., Morgado N., Longo N.S., Lipsky P.E. (2008). The NF-kappaB Canonical Pathway is Involved in the Control of the Exonucleolytic Processing of Coding Ends during V(D)J Recombination. J. Immunol..

[65] Nelson G.A., Jones T.A., Chesnut A., Smith A.L. (2002). Radiation-Induced Gene Expression in the Nematode Caenorhabditis Elegans. J. Radiat. Res..

[66] Alwood J.S., Shahnazari M., Chicana B., Schreurs A.S., Kumar A., Bartolini A., Shirazi-Fard Y., Globus R.K. (2015). Ionizing Radiation Stimulates Expression of Pro-Osteoclastogenic Genes in Marrow and Skeletal Tissue. J. Interferon Cytokine Res..

[67] Josson S., Xu Y., Fang F., Dhar S.K., St Clair D.K., St Clair W.H. (2006). RelB Regulates Manganese Superoxide Dismutase Gene and Resistance to Ionizing Radiation of Prostate Cancer Cells. Oncogene.

[68] Xu Y., Fang F., St Clair D.K., Josson S., Sompol P., Spasojevic I., St Clair W.H. (2007). Suppression of RelB-Mediated Manganese Superoxide Dismutase Expression Reveals a Primary Mechanism for Radiosensitization Effect of 1alpha,25-dihydroxyvitamin D(3) in Prostate Cancer Cells. Mol. Cancer Ther..

[69] Xu Y., Fang F., St Clair D.K., Sompol P., Josson S., St Clair W.H. (2008). SN52, A Novel Nuclear Factor-kappaB Inhibitor, Blocks Nuclear Import of RelB:p52 Dimer and Sensitizes Prostate Cancer Cells to Ionizing Radiation. Mol. Cancer Ther..

[70] Holley A.K., Xu Y., St Clair D.K., St Clair W.H. (2010). RelB Regulates Manganese Superoxide Dismutase Gene and Resistance to Ionizing Radiation of Prostate Cancer Cells. Ann. N. Y. Acad. Sci..

[71] Ray M., Yunis R., Chen X., Rocke D.M. (2012). Comparison of Low and High Dose Ionising Radiation Using Topological Analysis of Gene Coexpression Networks. BMC Genom..

[72] Fujimoto Y., Tedder T.F. (2006). CD83: A Regulatory Molecule of the Immune System with Great Potential for Therapeutic Application. J. Med. Dent. Sci..

[73] McKinsey T.A., Chu Z., Tedder T.F., Ballard D.W. (2000). Transcription Factor NF-kappaB Regulates Inducible CD83 Gene Expression in Activated T Lymphocytes. Mol. Immunol..

[74] Yang Y., Wang X., Moore D.R., Lightfoot S.A., Huycke M.M. (2012). TNF-alpha Mediates Macrophage-Induced Bystander Effects through Netrin-1. Cancer Res..

[75] Onizawa M., Nagaishi T., Kanai T., Nagano K., Oshima S., Nemoto Y., Yoshioka A., Totsuka T., Okamoto R., Nakamura T. (2009). Signaling Pathway via TNF-alpha/NF-kappaB in Intestinal Epithelial Cells may be Directly Involved in Colitis-Associated Carcinogenesis. Am. J. Physiol. Gastrointest. Liver Physiol..

[76] Chan A.T., Ogino S., Giovannucci E.L., Fuchs C.S. (2011). Inflammatory Markers are Associated with Risk of Colorectal Cancer and Chemopreventive Response to Anti-Inflammatory Drugs. Gastroenterology.

[77] Thommesen L., Laegreid A. (2005). Distinct Differences between TNF Receptor 1- and TNF Receptor 2-mediated Activation of NFkappaB. J. Biochem. Mol. Biol..

[78] Chen G., Goeddel D.V. (2002). TNF-R1 Signaling: A Beautiful Pathway. Science.

[79] Meng Z., Lou S., Tan J., Xu K., Jia Q., Zheng W. (2012). Nuclear Factor-kappa B Inhibition can Enhance Apoptosis of Differentiated Thyroid Cancer Cells Induced by 131I. PLoS ONE.

[80] Baggiolini M., Walz A., Kunkel S.L. (1989). Neutrophil-Activating Peptide-1/interleukin 8, a Novel Cytokine that Activates Neutrophils. J. Clin. Inv..

[81] Stein B., Baldwin A.S. (1993). Distinct Mechanisms for Regulation of the Interleukin-8 Gene Involve Synergism and Cooperativity between C/EBP and NF-kappa B. Mol. Cell. Biol..

[82] Wu G.D., Lai E.J., Huang N., Wen X. (1997). Oct-1 and CCAAT/enhancer-binding Protein (C/EBP) Bind to Overlapping Elements within the Interleukin-8 Promoter. The role of Oct-1 as a Transcriptional Repressor. J. Biol. Chem..

[83] Stewart J., Ko Y.H., Kennedy A.R. (2007). Protective Effects of L-Selenomethionine on Space Radiation Induced Changes in Gene Expression. Radiat. Environ. Biophys..

[84] Walenta S., Mueller-Klieser W. (2016). Differential Superiority of Heavy Charged-Particle Irradiation to X-rays: Studies on Biological Effectiveness and Side Effect Mechanisms in Multicellular Tumor and Normal Tissue Models. Front. Oncol..

[85] Tschachojan V., Schroer H., Averbeck N., Mueller-Klieser W. (2014). Carbon Ions and X-rays Induce Proinflammatory Effects in 3D Oral Mucosa Models with and without PBMCs. Oncol. Rep..

[86] Parihar V.K., Maroso M., Syage A., Allen B.D., Angulo M.C., Soltesz I., Limoli C.L. (2018). Persistent Nature of Alterations in Cognition and Neuronal Circuit Excitability after Exposure to Simulated Cosmic Radiation in Mice. Exp. Neurol..

[87] Parihar V.K., Allen B.D., Caressi C., Kwok S., Chu E., Tran K.K., Chmielewski N.N., Giedzinski E., Acharya M.M., Britten R.A. (2016). Cosmic Radiation Exposure and Persistent Cognitive Dysfunction. Sci. Rep..

[88] Choudhury A., Cuddihy A., Bristow R.G. (2006). Radiation and New Molecular Agents Part I: Targeting ATM-ATR Checkpoints, DNA Repair, and the Proteasome. Semin. Radiat. Oncol..

[89] Ao N., Chen Q., Liu G. (2017). The Small Molecules Targeting Ubiquitin-Proteasome System for Cancer Therapy. Comb. Chem. High Throughput Screen..

[90] Salminen A., Lehtonen M., Suuronen T., Kaarniranta K., Huuskonen J. (2008). Terpenoids: Natural Inhibitors of NF-kappaB Signaling with Anti-Inflammatory and Anticancer Potential. Cell. Mol. Life Sci..

[91] Jain H., Dhingra N., Narsinghani T., Sharma R. (2016). Insights into the Mechanism of Natural Terpenoids as NF-kappaB Inhibitors: An Overview on Their Anticancer Potential. Exp. Oncol..

[92] Hellweg C.E., Langen B., Klimow G., Ruscher R., Schmitz C., Baumstark-Khan C., Reitz G. (2009). Up-Stream Events in the Nuclear Factor κB Activation Cascade in Response to Sparsely Ionizing Radiation. Adv. Space Res..

[93] Burdelya L.G., Krivokrysenko V.I., Tallant T.C., Strom E., Gleiberman A.S., Gupta D., Kurnasov O.V., Fort F.L., Osterman A.L., Didonato J.A. (2008). An Agonist of Toll-Like Receptor 5 Has Radioprotective Activity in Mouse and Primate Models. Science.

[94] Graham F.L., Smiley J., Russell W.C., Nairn R. (1977). Characteristics of a Human Cell Line Transformed by DNA from Human Adenovirus Type 5. J. Gen. Virol..

[95] Durantel F., Balanzat E., Cassimi A., Chevalier F., Ngono-Ravache Y., Madi T., Poully J.-C., Ramillon J.M., Rothard H., Ropars F. (2016). Dosimetry for Radiobiology Experiments at GANIL. Nucl. Instrum. Methods Phys. Res. Sect. A: Accel. Spectrom. Detect. Assoc. Equip..

[96] Wulf H., Kraft-Weyrather W., Miltenburger H.G., Blakely E.A., Tobias C.A., Kraft G. (1985). Heavy-Ion Effects on Mammalian Cells: Inactivation Measurements with Different Cell Lines. Radiat. Res. Suppl..

